# Molecular Dynamics Simulations Reveal Proton Transfer Pathways in Cytochrome C-Dependent Nitric Oxide Reductase

**DOI:** 10.1371/journal.pcbi.1002674

**Published:** 2012-08-30

**Authors:** Andrei V. Pisliakov, Tomoya Hino, Yoshitsugu Shiro, Yuji Sugita

**Affiliations:** 1 Theoretical Molecular Science Laboratory, RIKEN Advanced Science Institute, Wako-shi, Saitama, Japan; 2 RIKEN SPring-8 Center, Sayo, Hyogo, Japan; 3 Computational Biophysics Research Team, RIKEN Advanced Institute for Computational Science, Chuo-ku, Kobe, Japan; 4 Laboratory for Biomolecular Function Simulation, RIKEN Quantitative Biology Center, Chuo-ku, Kobe, Japan; Wellcome Trust Sanger Institute, United Kingdom

## Abstract

Nitric oxide reductases (NORs) are membrane proteins that catalyze the reduction of nitric oxide (NO) to nitrous oxide (N_2_O), which is a critical step of the nitrate respiration process in denitrifying bacteria. Using the recently determined first crystal structure of the cytochrome *c*-dependent NOR (cNOR) [Hino T, Matsumoto Y, Nagano S, Sugimoto H, Fukumori Y, et al. (2010) Structural basis of biological N2O generation by bacterial nitric oxide reductase. Science 330: 1666–70.], we performed extensive all-atom molecular dynamics (MD) simulations of cNOR within an explicit membrane/solvent environment to fully characterize water distribution and dynamics as well as hydrogen-bonded networks inside the protein, yielding the atomic details of functionally important proton channels. Simulations reveal two possible proton transfer pathways leading from the periplasm to the active site, while no pathways from the cytoplasmic side were found, consistently with the experimental observations that cNOR is not a proton pump. One of the pathways, which was newly identified in the MD simulation, is blocked in the crystal structure and requires small structural rearrangements to allow for water channel formation. That pathway is equivalent to the functional periplasmic cavity postulated in *cbb*_3_ oxidase, which illustrates that the two enzymes share some elements of the proton transfer mechanisms and confirms a close evolutionary relation between NORs and C-type oxidases. Several mechanisms of the critical proton transfer steps near the catalytic center are proposed.

## Author Summary

Denitrification is an anaerobic process performed by several bacteria as an alternative to aerobic respiration. A key intermediate step is catalyzed by the nitric oxide reductase (NOR) enzyme, which is situated in the cytoplasmic membrane. Proton delivery to the catalytic site inside NOR is an important part of its functioning. In this work we use molecular dynamics simulations to describe water distribution and to identify proton transfer pathways in cNOR. Our results reveal two channels from the periplasmic side of the membrane and none from the cytoplasmic side, indicating that cNOR is not a proton pump. It is our hope that these results will provide a basis for further experimental and computational studies aimed to understand details of the NOR mechanism. Furthermore, this work sheds light on the molecular evolution of respiratory enzymes.

## Introduction

Bacterial denitrification is one of the examples of anaerobic respiration in which nitrate (NO_3_^−^) is stepwisely reduced to dinitrogen (N_2_) [1]–[3]. During denitrification, the key intermediate step of the reduction of nitric oxide (NO) to nitrous oxide (N_2_O) is catalyzed by a membrane-bound enzyme nitric oxide reductase (NOR) according to the following scheme: 2NO+2e^−^+2H^+^→N_2_O+H_2_O. Bacterial NORs perform fundamental chemistry and are the largest source of N_2_O, a greenhouse gas and an ozone-depleting substance, released into the atmosphere [1]. This enzyme also has an important role in the evolution of the respiratory system. NOR belongs to the superfamily of O_2_-reducing heme-copper oxidases (HCOs) and is believed to be evolutionary linked to a proton pump cytochrome *c* oxidase (CcO). Both enzymes may have evolved from a common ancestor [2]. The ancestral oxidase was probably involved in NO reduction, but later switched to oxygen reduction and additionally acquired the ability of proton pumping, although this issue is still open to debate [3]–[9].

After the structure of CcO was solved more than a decade ago [10], [11], that system became the focus of numerous experimental studies, which produced a number of X-ray structures from different organisms and a wealth of mutational, biochemical and spectroscopic data, as well as theoretical and simulation ones (for recent reviews, see refs. [12]–[14]). In contrast, the information about NORs was limited, but the first structure of cytochrome *c*-dependent NOR (cNOR) from *Ps. aeruginosa* has been recently determined by Shiro and co-workers [15], and that provides a basis for studies aimed at describing the mechanism of NO reduction at the atomic level. cNOR consists of two subunits, NorB and NorC, and contains four redox active metal centers, namely hemes *b*, *b*_3_ and *c* and a non-heme iron (Fe_B_). The latter and the iron of heme *b*_3_ form the binuclear (BN) center, a site of the NO reduction. The crystal structure revealed that Fe_B_ has three His and one Glu ligands and that a tightly bound Ca^2+^ ion is bridging hemes *b* and *b*_3_. Although the function of Ca^2+^ is not yet fully clear, it is interesting to note that it has the same binding position as in a recently determined structure of the microaerobic respiratory enzyme *cbb*_3_ oxidase [16], which is a C-type HCO able to reduce NO to N_2_O in low-oxygen conditions [17], [18].

For the NO reduction reaction protons have to be delivered to the BN center, which is buried inside membrane. Previous experiments with the whole-cell [19] and liposome-reconstituted [20], [21] cNORs demonstrated that protons utilized in the catalytic reaction are taken up (on a ms timescale) from the periplasmic side (i.e. the same side as electrons), which suggests that the NO reduction reaction is non-electrogenic and therefore cNOR is not a proton pump. In order to explain the functioning of cNOR, it is necessary to understand the detailed mechanism of the proton delivery to the BN center. Since proton transfer (PT) can occur efficiently only when the donor and acceptor groups are immediately close to each other, the long-distance proton translocations in proteins (e.g. proton pumping across the membrane or proton delivery from the bulk to the buried active site) require specialized proton-conducting pathways, which involve protein ionizable groups and intermediate water molecules as proton-binding sites (see e.g. refs. [22]–[28]).

Analysis of the cNOR crystal structure yielded two independent H-bonded networks, designated as Channels 1 and 2, which are formed by the resolved water molecules and the charged/polar residues [15]. These channels were proposed as potential PT pathways. However, since X-ray crystallography provides only static snapshots of the protein structure, which are averaged over many unit cells, in general such structures even at high resolution show only a few water molecules (at the most stable positions) inside the protein but miss many dynamic ones. The proposed proton pathways did not provide a continuous connection from the surface to the active site (i.e. protonic “gaps” were present), and in particular the pathways near the catalytic center, where no water molecules were resolved, remained elusive. As we mentioned, in such situations the connectivity is expected to come from the intervening water molecules. Thus, water in cNOR could play a very important role in the enzyme function and has to be fully characterized.

Molecular dynamics (MD) simulations of membrane proteins within an explicit membrane/solvent environment (for some recent works and reviews see refs. [28]–[35]) can provide important information about the water dynamics, such as a “real” level of hydration and specific water positions inside the protein, and are most valuable in the cases when the water/PT channels are not yet described at the atomic level. For example, MD simulations have been recently used to explore the water dynamics in different regions in CcO and greatly contributed to the understanding of the details of PT channels in that enzyme [36]–[42]. We note that a study with an explicit membrane/solvent by Olkhova *et al.* [39] suggested a large number of water molecules within the PT channels in CcO, in contrast to simulations which utilized different kinds of reduced models or truncated systems.

In this work we performed MD simulations of cNOR. We focused on the water dynamics, with the aim to identify the water channels and H-bonded networks that could serve as pathways for the proton delivery to the active site. The obtained information will be important for further elucidation of the mechanisms of the proton translocation and NO reduction in cNOR.

## Methods

**Figure 1:**
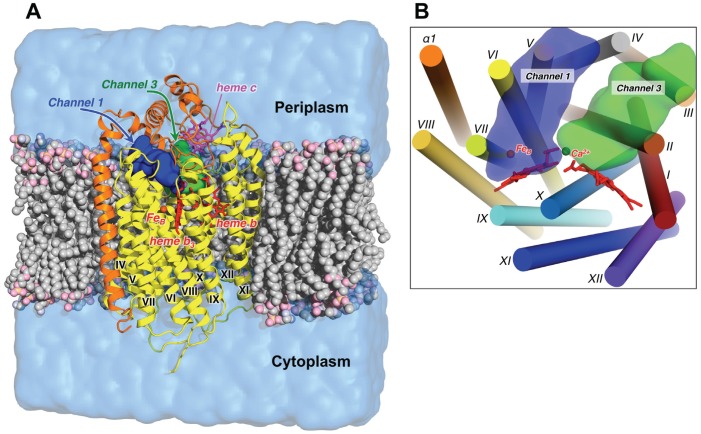
(**A**) A setup of a molecular dynamics simulation. A simulation system is composed of *Ps. aeruginosa* cNOR (PDB ID: 3O0R) [15] embedded in a POPE lipid bilayer membrane and a water solvent box. NorB and NorC subunits are colored in yellow and orange. The heme *b*, *b*_3_ (red) and *c* (magenta) groups are shown as sticks and Fe_B_ (orange) and Ca^2+^ (magenta) ions as spheres. Lipid molecules are shown in a sphere representation and water as a transparent light-blue surface. (For clarity of the figure, only some lipid molecules and a part of the water box are shown.) The blue and green surfaces show the positions of two potential proton pathways from the periplasmic side of the membrane, Channels 1 and 3, which are discussed in this work. (**B**) Arrangement of the cNOR membrane-spanning helices and positions of Channels 1 and 3 as viewed from the periplasm. Transmembrane helices of the NorB subunit are indicated with Roman numerals (I–XII) and a helix of the NorC subunit as α1. (The secondary structure assignment is taken from the crystal structure [15].) Molecular graphics were produced using PyMOL [75].

We performed an all-atom MD simulation of cNOR in the explicit lipid/water environment (Figure 1a). The details of the system setup and simulation and analysis protocols are provided in the Text S1. Briefly, the initial system was prepared from a 2.7 Å resolution crystal structure of the cNOR from *Ps. aeruginosa* (PDB ID 3O0R) [15]. A simulation system is shown on Figure 1a: cNOR was embedded into the pre-equilibrated POPE (palmitoyl-oleoyl-phosphatidylethanolamine) lipid bilayer membrane and a solvent box of water molecules. The total size of the simulation system was ∼110,000 atoms. The main purpose of introducing the lipid bilayer in MD simulation is to model cNOR *in situ*, i.e. in the environment as close to its natural as possible. POPE is the major lipid component of bacterial membranes [43]. Explicit membrane provides additional stability to the protein in MD simulations and allows a correct description of the protein-solvent and protein-lipids interactions.

MD simulations were carried out in NAMD [44] with the CHARMM force field [45], [46]. After minimization and equilibration parts, production runs were performed at a constant temperature, pressure, and surface area (NPAT ensemble) for 300 ns, providing reasonable conformational sampling of the protein. Stability of the simulated protein-membrane complex was assessed from the analysis of several parameters along the MD trajectory (Figure S1). The root-mean-square deviation (RMSD) of the helical C_α_ atoms is below 2 Å while RMSD of C_α_ atoms in a transmembrane (TM) region is ∼1.2 Å. The RMS fluctuations (RMSFs) calculated for each residue also illustrate that the TM region is very stable while the outer and inner domains exhibit, as expected, larger motions. Finally, the area/lipid, which was calculated using the Voronoi analysis tool [47], remains close to the experimental value for the POPE lipids [48], also indicating a stable simulation of the protein-membrane complex.

## Results/Discussion

### Periplasmic Channel 1

**Figure 2:**
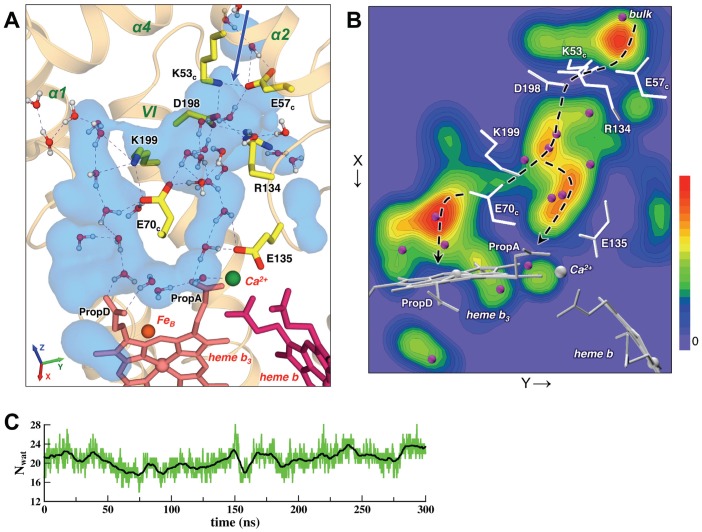
Channel 1, the proposed proton pathway. (**A**) A representative MD snapshot (at ∼100 ns) of the Channel 1 region. The pathway starts near Lys53_c_, Glu57_c_ and Asp198 (the entrance from the bulk is indicated by a blue line) and leads to a water cluster near the heme *b*_3_ propionates via a number of conserved charged residues and water molecules (see details in the text). Hemes *b* (dark-red) and *b*_3_ (pink) and the charged residues lining the channel (colored by atom types, with carbons in yellow) are shown as sticks, Fe_B_ (orange) and Ca^2+^ (green) ions as spheres, and water molecules present in or near the channel are shown in ball-and-stick representation (red/white). Important helices are labeled (green). The dashed lines indicate the hydrogen bonds within the forming continuous H-bonded networks, which connect the periplasmic surface with the propionates of heme *b*_3_. In the MD simulation the Channel 1 interior region is very well hydrated, as illustrated by the water density averaged over 300 ns (a transparent light-blue isosurface shown at 25% occupancy). Figure reveals two branches of the water channel at both sides of Glu70_c_. (**B**) Water density in Channel 1 shown as a 2D contour map (a projection on the XY-plane was obtained by summing the water density in the Channel 1 region over the vertical Z-axis). Different colors correspond to water residence values, ranging from red (high water residence) to blue (low water residence). Positions of the Channel 1 residues and crystallographic waters in the cNOR X-ray structure are shown for reference as white sticks and purple spheres, respectively. Two branches of the potential proton pathway (indicated by the dashed black lines) go through high-occupancy water sites, which in general superimpose well with the positions of crystallographic waters. (**C**) Number of water molecules in the Channel 1 region in the MD simulation; black line represents a running average over 30 data points. Water molecules within 4.5 Å of the channel residues were selected.

One of the proposed PT pathways (Channel 1 in Figure 3 in ref. [15]) goes through a large hydrophilic region, which is located on the periplasmic side of the enzyme at the interface of a TM region (NorB subunit) and an outer soluble domain (NorC subunit) (Figure 1). Four residues, namely Glu135, Asp198, Lys53_c_, and Glu57_c_, were designated as Channel 1 [15]. [Subscript “c” indicates residues of the NorC subunit, while residues of the NorB subunit are numbered without additional subscripts.] In MD simulations we observed that Channel 1 indeed connects the protein surface to propionates of heme *b*_3_ (the distance ∼16 Å) via a number of ionizable residues and water molecules and supports formation of the H-bonded networks (see below). Our analysis provides important additional details about Channel 1 (Figure 2a). MD results indicate that the following three residues participate in the HB networks in that region: Arg134, Lys199, and Glu70_c_, and therefore they have to be included in Channel 1. All seven ionizable residues are highly conserved in cNORs. Together, they line up a large hydrophilic channel, and their sidechains assist in the formation of the H-bonded water chains.

Channel 1 has a connection to the bulk solvent between two helices, TM VI of NorB (with Asp198 and Lys199) and α2 of NorC (with Lys53_c_ and Glu57_c_). The entrance site formed by the amino acids Glu57_c_, Lys53_c_, and Arg134 (Figures 2a and S2) remains rigid due to three stable salt bridges: Glu57_c_-Lys53_c_, Arg134-Asp198, and Lys53_c_-Asp198 (Figure S3). These residues partially block water influx. However, water molecules still occasionally cross through that site, and thus can serve as intermediate proton sites (Figure S4). Also, the dynamic HB networks involving sidechains of Glu57_c_ and Asp198 and waters at both sides of the entrance, i.e. in the bulk and inside the Channel 1 cavity, are observed at any time of MD trajectory. Therefore it is possible that one of these residues could be directly involved in PT by picking up protons from the bulk and releasing them to the water chain inside Channel 1. The mutagenesis experiments with *P. denitrificans* cNOR [Pia Ädelroth, unpublished data] showed the importance of Asp185 (equivalent to Asp198 in *Ps. aeuginosa* cNOR) for the enzymatic activity and proton uptake, and provide partial support to this proposal.

From the entrance region the proton pathway proceeds further through the dynamic water chains. Water channels in cNOR have “irregular” shape and lack simple symmetry (like, e.g. straight TM channels in aquaporins or ion channels). Therefore to perform meaningful statistical analysis in each region we selected water molecules within a reasonable distance cutoff (typically 4.5 Å) near the sidechains of the pathway's residues. We verified that with such definition water molecules “inside the pathway” were not skipped. Our calculations show that Channel 1 is very well hydrated: in MD simulation ∼20 water molecules are observed in this hydrophilic region (Figure 2c), which is higher than ∼12 molecules resolved in the X-ray structure. This result can be explained by the presence of mobile water molecules, which were not resolved in X-ray crystallography.

To provide some quantitative representation, we have calculated volume occupied by water molecules during simulation (“water density”, see e.g. refs. [49], [50]) in different regions of cNOR. Figure 2 illustrates water spatial distribution in Channel 1 as obtained from MD simulation, showing both a 3D water volume map (Figure 2a, isosurface at 25% occupancy) and a 2D contour plot (Figure 2b, an XY-plane projection of the water density; see figure caption for details). A few observations can be made from these figures. (i) Water density representation shows the extent of the hydrophilic regions and confirms a stable connection from the bulk to the active site heme. (ii) Water molecules form an extensive water cluster between propionates of heme *b*_3_ (PropA and PropD). [Please note that compared to the previously reported cytochrome c oxidase structures the active site heme in cNOR, i.e. heme *b3*, is flipped and the order of the propionate groups A and D is different.] (iii) After the entrance region, the channel goes into a water-filled cavity. An important finding is that further the pathway splits into two branches: one path leads via a water chain (5–6 water molecules) directly to PropA, while another – via Glu70_c_ and a short chain (2–3 water molecules) at the other side of that residue – to PropD. The terminal region of both paths is the water cluster near heme *b_3_* propionates. The existence of two branches in Channel 1 could be observed in the MD simulation, but was not evident from the static X-ray structure. This feature provides a possibility of PT over different pathways and probably adds to the robustness of the proton uptake via Channel 1. (iv) When water density is plotted at a higher occupancy level, one obtains positions of the water sites that are occupied almost permanently during the simulation. One example is a crystallographic water molecule, Wat65, which remains bound near Ca^2+^ for the entire MD trajectory. Such “permanent” water sites in general superimpose well with the positions of waters resolved in the X-ray structure (indicated by purple spheres on Figure 2b).

Figure 2a also presents a typical configuration of the H-bonded networks forming in Channel 1, while Video S1 and multiple MD snapshots on Figure S5 illustrate their time-dependent dynamics. The average lifetime of a hydrogen bond (HB) in the water chains is in the ps range due to rotating and/or moving water molecules. It can be seen that water molecules in Channel 1 have high mobility and exchange rates and, as a result, the forming H-bonded networks are constantly “fluctuating” (similar to the H-bonded networks in CcO [39], [40]). Continuous HB paths between the bulk and heme *b_3_* propionates do form, and their consistency is limited by the intervening water chains, namely a chain from Asp198 to Glu135 (probability ∼25–35%) and a chain from Asp198 to Glu70_c_ (probability >60%). But it is important that such connections are forming at all times, and thus can assist efficient proton translocation [51]. Participation of the Channel 1 residues in the H-bonded networks can be assessed quantitatively by calculating the number of surrounding water molecules and formed HBs (Table S1). In particular, these results, in addition to visual analysis, suggest that Glu70_c_ could play an important role in the proton uptake process. It is desirable to verify its involvement in the PT pathway by site-directed mutagenesis experiments.

### Periplasmic Channel 2

**Figure 3:**
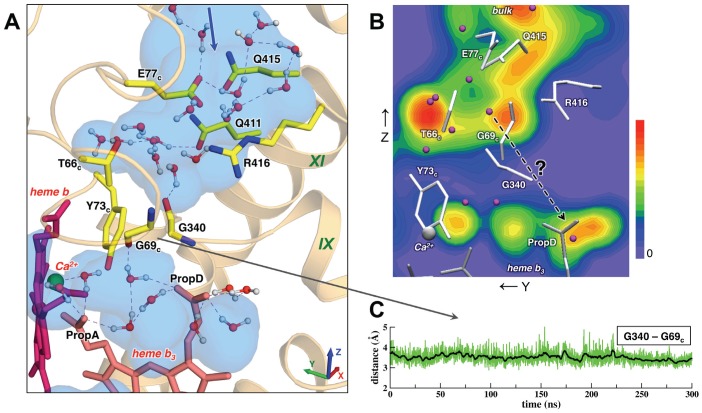
The MD simulation does not support the previously suggested Channel 2 as a possible proton uptake pathway. (**A**) A representative MD snapshot (at ∼170 ns) and the calculated water density (the color coding is as in Figure 2a) show that water molecules in the upper hydrophilic region are separated from the water cluster near heme *b*_3_ by two loops (see details in the text). (**B**) Water density shown as a 2D contour plot (a projection on the YZ-plane; the color coding is as in Figure 2b) and as a density profile along Z-coordinate. (**C**) Gly340-Gly69_c_ distance time series from the MD simulation.

From the inspection of the X-ray structure, Hino *et al.* identified another cavity, which contains many crystallographic waters, and proposed it as a second possible proton-conducting pathway (Channel 2 in Figure 3 in ref. [15]). In MD simulation we observed a large hydrophilic region formed by the residues Arg416, Thr66_c_, Glu77_c_, Gln411, and Gln415, with an exit to the bulk beyond the latter (Figure 3a). On average, there are 10 to 12 water molecules in the cavity. However, we found that water molecules from this cavity cannot pass to the water cluster near heme *b_3_*, and further to the active site. Two loops, and more specifically two glycine residues Gly340 and Gly69_c_, are in close contact en route to heme *b_3_* and, together with a ring of Tyr73_c_, disrupt a possible water chain. A close steric contact between two loops remains for the entire length of the MD trajectory, as evidenced by the Gly-Gly distance (Figure 3c), which stays around 3.5–4 Å (i.e. similar to the distance in the crystal structure). Water densities (Figures 3a and b) clearly show a wide gap with no substantial density between the upper hydrophilic cavity and the water cluster. We do not completely rule out a possibility that mobile water molecules can occasionally cross the gap region; however, no such crossings or continuous HB networks were observed in 300 ns. Moreover, the proton translocation through the region with no polar/charged residues or water molecules would encounter high activation barriers. Thus, our results do not support the previously proposed Channel 2 as an alternative pathway for proton delivery to the active site. The exact functional role of this hydrophilic cavity in cNOR is not clear.

### Periplasmic Channel 3

**Figure 4:**
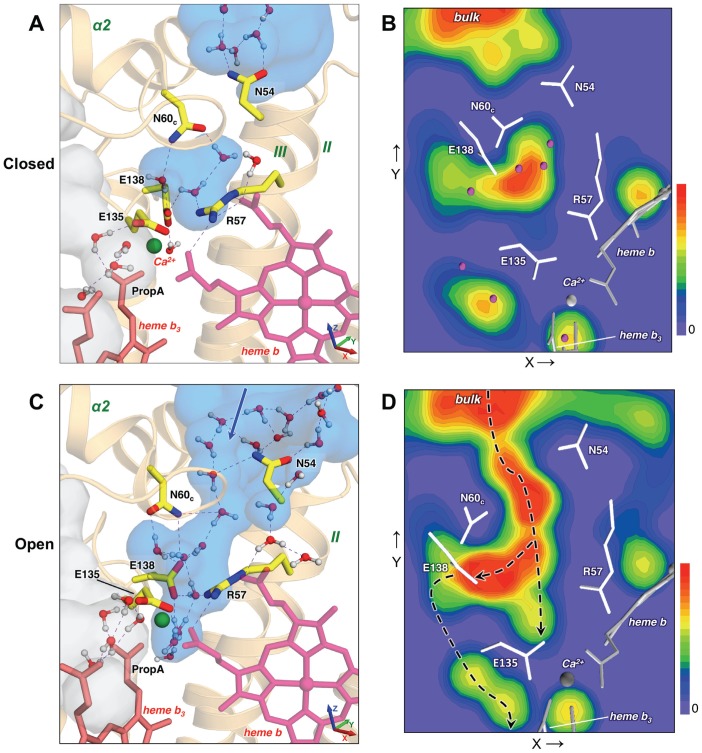
New plausible proton pathway, Channel 3, revealed by the MD simulations. (**A**) A representative configuration (MD snapshot at ∼25 ns) of the Channel 3 region when the Asn54-Asn60_c_ gate is closed and the internal hydrophilic cavity between Asn60_c_, Glu138 and Arg57 has no connection to the outside bulk. (The color coding is as in Figure 2a; the water density was averaged over the first 165 ns.) (**B**) Water density in the Channel 3 region averaged over the first 165 ns, shown as a 2D contour map (a projection on the XY-plane; the color coding is as in Figure 2b.) (**C**) A representative configuration (MD snapshot at ∼200 ns) when the gating residues, Asn54 and Asn60_c_, move away from each other. The water density, which was averaged over the interval 165–225 ns, when the gate is open, shows a newly formed water channel. The dynamic H-bonded water chains connect the bulk to the two important residues, Glu138 and Glu135, and can facilitate PT toward the active site. The new suggested pathway is spatially separated from Channel 1, part of which is shown for reference as a light-gray surface to the left of heme *b*_3_. (**D**) Same as in B, but the water density was averaged over 165–225 ns. When the Asn-Asn gate is open, the continuous water distribution from the bulk up to Glu135/Ca^2+^ site and Glu138 is observed. The possible water-mediated PT pathway is indicated by the dashed black lines, with the path via Glu138 to the water sites near the BN center being more plausible (see Figure 7 and discussion therein).

A careful analysis of MD trajectories revealed another plausible proton pathway, which we designated as the (periplasmic) Channel 3 (see Figures 1 and 4). This pathway involves the residues Glu135, Glu138, Arg57, Asn54, and Asn60_c_. The first three are highly conserved in all NORs and oxidases, while Asn54 is conserved in cNORs. In the X-ray structure, three water molecules are resolved in a cavity formed by these residues. During the initial part of the MD simulation this region has no connection to the periplasmic surface (Figures 4a, b). The calculated water density clearly shows that the cavity is completely separated from the bulk solvent and that two asparagine residues, Asn54 and Asn60_c_, effectively work as a gate, blocking water access from the outside.

**Figure 5:**
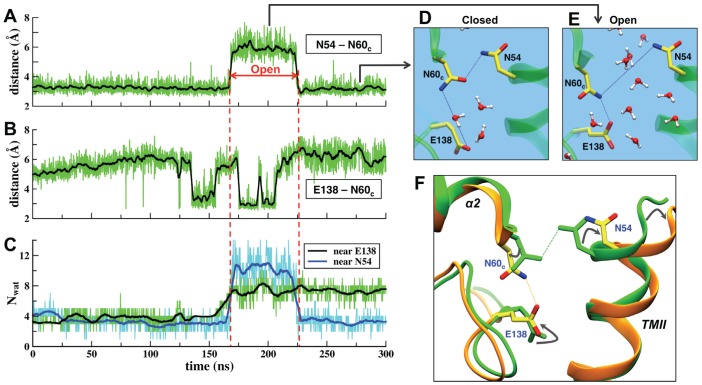
Gating of Channel 3. (**A–C**) The time series of the minimal distances between Asn54-Asn60_c_ and Glu138-Asn60_c_ residues and the number of water molecules near sidechains of Glu138 and Asn54 in the MD simulation. The vertical red dashed lines indicate the interval when the Asn-Asn gate is open and the water channel is formed. Glu138 remains well hydrated even after the gate closing. (**D–E**) Close-up views of the gate region for the closed and open cases. (**F**) An overlay of the closed (green) and open (orange) structures of the Channel 3 gate region. The conformational changes associated with the gate opening involve rotation of the sidechains of Asn54, Asn60_c_ and Glu138 and tilting of the helix TM II.

However, after ∼165 ns in the MD simulation the Asn54-Asn60_c_ gate opens and a new water channel is formed (Figures 4c, d). A continuous water density then extends up to two important residues, Glu135 and Glu138, and the H-bonded networks involving mobile water molecules and amino acid sidechains readily form. The number of waters in the hydrophilic region, and in particular around the sidechain of Glu138, significantly increases with the gate opening and remains high even after the gate closes back (Figure 5c). Figure 5 also shows minimal distances between the Asn54-Asn60_c_ and Glu138-Asn60_c_ pairs in the MD simulation, along with the representative snapshots of the gate region. Clearly, the gate is closed when two Asn are H-bonded. Sidechains of Glu138 and Asn60_c_ exhibit large-amplitude rotations (Figure S6), in particular Glu138 can take several conformations, and the initial event leading to the gate opening seems to be a rotation of Glu138 to the “up” position after ∼135 ns and the formation of a HB to Asn60_c_. Soon after that a strong HB between two Asn is broken. As a result, a helix TM II (with Asn54 on a top) slightly tilts away, and that opens water access to the internal cavity.

An overlay of the open and closed configurations (Figure 5f) shows that the required structural changes are rather small: two Asn move away only by a few Å, but that is enough to break a HB between them and to open access to the internal cavity for water molecules from the outside. The gate is open for ∼60 ns, after which the HB between Asn60_c_ and Asn54 is re-formed; the HB between Glu138 and Asn60_c_ breaks prior to that. The explicit gate opening/closing process and formation of the dynamic water chains in Channel 3 are illustrated by Video S2. We would like to emphasize that similar events were also observed in the extended simulation as well as in independent runs (Figure S7), indicating that such events can occur in cNOR on a 100-ns timescale, which is much shorter than the experimentally measured rate of the proton uptake (∼25 ms) [20], [21]. This suggests that such structural reorganizations due to protein fluctuations are feasible during catalysis in cNOR and that Channel 3, in principle, can provide a pathway for a water-mediated proton uptake. We propose to examine the role of Asn54 and Asn60_c_ in the Channel 3 gating by the mutagenesis experiments.

A newly found channel is consistent with previous experimental data. Two key residues, Glu135 and Glu138 (Glu122 and Glu125 in *P. denitrificans* cNOR), were shown by site-directed mutagenesis to be essential for the enzymatic activity and were proposed to be a part of the proton input pathway [52]–[54], though their exact positions predicted with the homology-based model (namely, on a protein outer surface) [21] turned out to be incorrect. With the cNOR structure available now, it is known that Glu135 is a ligand to Ca^2+^. That explains why its substitution with Asp still showed a level of activity close to the wild type (i.e. Ca^2+^ coordination was kept) while a substitution with Ala or Gln resulted in a loss of activity (most likely caused by a Ca^2+^ dissociation). The structural function of Glu135 also makes its direct participation in PT problematic: it is unlikely that Glu135 can get protonated or that the protons coming from the periplasm can be transferred through a densely packed region occupied by the Ca^2+^ ion and its ligands.

The substitution of Glu138 with Ala and Asp resulted in a loss of activity, while a mutation to Gln showed some, though significantly reduced activity [53], [54]. These results could indicate that the length of the sidechain is more important than retaining a negative carboxylic group at that position. The observation fits into the above suggested mechanism of the Channel 3 opening and “activation” of the proton pathway, which includes a Glu138 sidechain rotation to the “up” position to form a HB to Asn60_c_, thus helping to break a HB between two Asn.

In contrast to Glu135, Glu138 can actively participate in the PT process. A Glu122Asp mutation in *P. denitrificans* caused a significant pK_a_ shift of a presumed nearby proton donor group [54], and Glu138 seems to be the best candidate for that role. The proton pathway beyond Glu138 is also offered by our MD results. After the gate opening, Glu138 is well hydrated, with typically 5 to 8 water molecules near its sidechain (Figures 4c,d and 5c). We observed the H-bonded water chains leading from this site toward the water molecules bound near BN, thus avoiding the Ca^2+^ site (see the corresponding discussion below).

**Figure 6:**
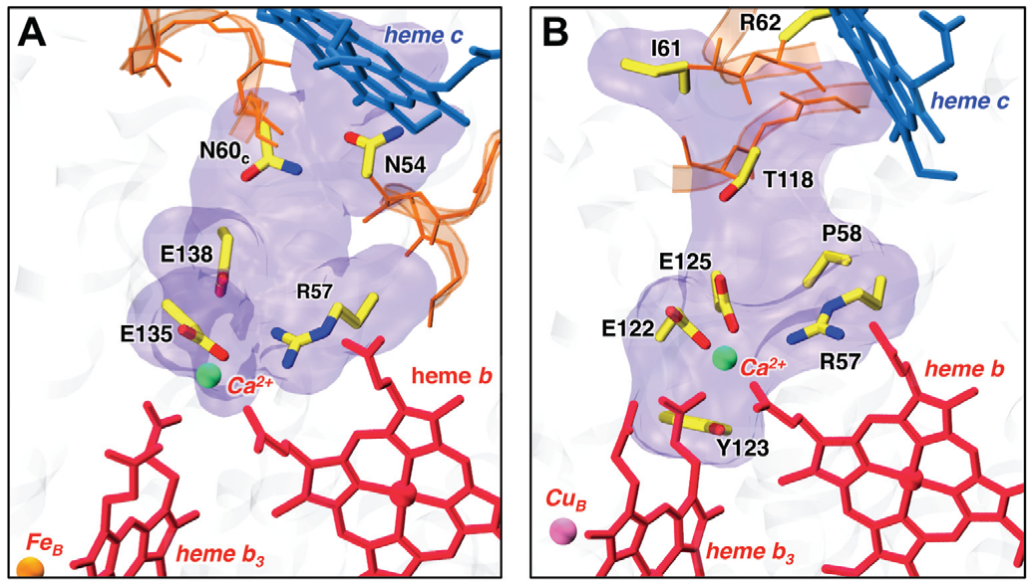
Comparison of the Channel 3 region in cNOR (A) and the periplasmic cavity in *cbb*_3_ oxidase (B). Crystal structures of cNOR from *Ps. aeruginosa* and *cbb*_3_ oxidase from *Ps. stutzeri* (PDB ID: 3MK7) [56] were aligned on hemes *b* and *b*_3_. The transparent blue surfaces indicate the positions of hydrophilic cavities. The central residues, which form the hydrophilic cavity (namely Glu135, Glu138, Arg57 in cNOR and Glu122, Glu125, Arg57 in *cbb*_3_ oxidase), are identical and highly conserved in the HCO superfamily. A calcium ion is located in a similar position between hemes.

A key finding is that the suggested novel channel in cNOR is equivalent to the putative PT pathway (the “periplasmic cavity”) in a recently determined structure of *cbb_3_* oxidase [16]: a comparison of two regions shows that their positions are identical (Figure 6). Moreover, the important residues which form this hydrophilic cavity, namely Glu135, Glu138 and Arg57 in cNOR and Glu122, Glu125, Arg57 in *cbb*_3_, are conserved. The periplasmic cavity in *cbb*_3_ oxidase was suggested to be an exit pathway of the pumped protons *or* a pathway for proton uptake from periplasm when the enzyme is involved in NO reduction [16]. The fact that for NO reduction *cbb_3_* uses protons from the periplasmic side of the membrane has been recently confirmed by the experimental work of Lee *et al.* [18]. We note that such cavity is not found in other structurally known HCOs and that *aa*_3_ oxidases (A-type HCOs) are incapable of NO reduction, while *ba*_3_ oxidases (B-type HCOs) can reduce NO but much slower than *cbb*_3_ [3], [5], [9]. The presence of a plausible PT pathway in the equivalent region in *cbb*_3_ oxidase is an additional argument for the functional importance of Channel 3 in cNOR. The finding that two enzymes likely have common elements of the PT mechanism, along with other common structural factors, such as the identical position of Ca^2+^, fits nicely into the phylogenetic pictures that draw C-type HCOs as the closest evolutionary relatives of NORs.

We have also analyzed the region equivalent to Channel 1 in the *cbb*_3_ structure [16]. It seems that the corresponding region cannot provide a pathway for proton translocation in *cbb*_3_ because: (i) some of the charged residues present in Channel 1 in cNOR, namely Lys199, Lys53_c_, Glu57_c_, and Glu70_c_, are either missing or located far away in *cbb*_3_, (ii) a coil with several hydrophobic residues is located in the central part of that region and splits it into two parts; the water distribution is disconnected too [to be published], (iii) a second Ca^2+^ site is located at the position equivalent to the entrance to Channel 1 in cNOR and most likely blocks proton transfer.

### PT pathways near the active site

We have shown that Channel 1 and Channel 3 can connect the periplasmic surface to the region near heme *b*_3_. Its propionates together with a nearby water cluster and Glu138 are the likely intermediate proton acceptor groups. (It is less likely that PropA can get protonated since it serves as a ligand to Ca^2+^.) It is worth mentioning that in CcO one of the active site heme propionates is thought to be the likely proton loading site for the pumped protons [55]–[57]. The idea about the functional importance of protonated water clusters inside proteins is also not new. For example, in CcO a protonated water cluster was suggested as a proton storage site in the D-channel [58], while in bacteriorhodopsin a protonated water cluster is a presumed proton release group [59], [60].

**Figure 7:**
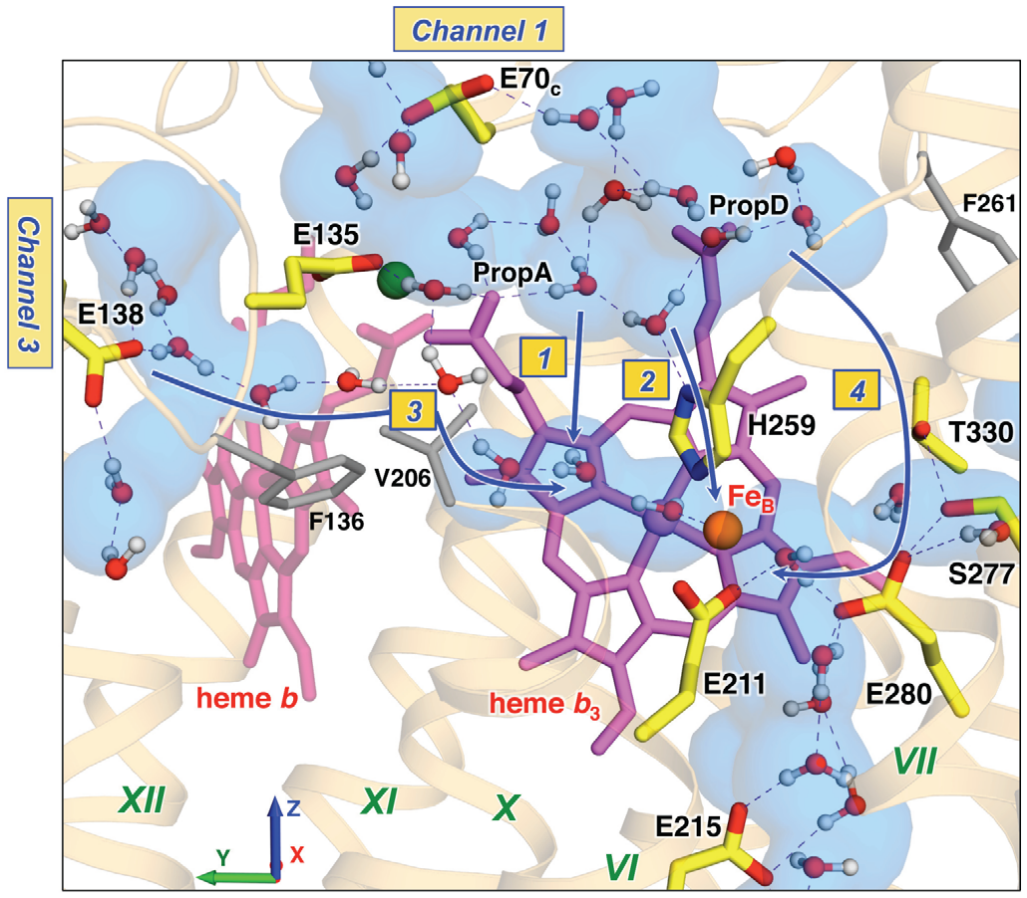
Possible PT pathways near the active site. A representative configuration (MD snapshot at ∼240 ns) of the water molecules and transiently forming H-bonded water chains in the region around the BN center. The important residues are shown. (The color coding is as in Figure 2a; the water density in the region, including the terminal parts of Channels 1 and 3, was averaged over 300 ns.) Possible paths for the final PT steps to the BN center are indicated by blue lines and marked with numbers 1 to 4 (see discussion in the text).

An important question is how protons are delivered to the catalytic center when they are needed for the NO reduction, i.e. what are the structural elements critical for the final PT steps? The distance (>8 Å) is still long for direct PT, but no water molecules were resolved in the vicinity of the BN center. So the further proton path was not clear from the X-ray structure, and intermediate water molecules are expected to play important role. In a working enzyme, water will be produced at the active site as a byproduct of the catalytic NO reduction. In contrast to the crystal structure, the MD simulation reveals the presence of water molecules near the BN center (Figure S8) and describes their distribution (Figure 7). The exchange rate of waters is much lower compared to the channels discussed above. Water molecules are found persistently at several positions and keep these positions for 20–50 ns or longer (Figure S9); such water molecules might serve as intermediate proton sites. Figure 7 depicts a representative configuration of water molecules in that region, along with the calculated water density (see also Figure S10). It can be seen that one permanent water site is located between two irons of the BN center (i.e. where NO ligands will bind during the enzymatic cycle), another corresponds to the water molecule bound between Fe_B_ and Glu280, and two more water sites are located between Fe_B_ and PropA. It is interesting that in a recent high-resolution structure of *Th. thermophilus ba*_3_ oxidase [61] two water molecules were resolved at the identical positions.

Analysis of the water dynamics and distribution offers several possible paths for the final PT steps to the BN center (Figure 7):

1. *from the water cluster to the water sites near BN*. The waters in these two regions come as close as ∼4–5 Å, and a single mobile water molecule can provide a protonic connection.
2. *from the water cluster or PropD to BN via His259*. His259 is a top ligand of Fe_B_ and most of the time is H-bonded to the water molecule from the cluster (this HB exists in 63% of simulated time). Several theoretical studies of CcO suggested that equivalent histidine, which is a top ligand of the Cu_B_, plays the role of the pump element [62].
3. *from Glu138 to the water sites near BN*. Bridging water molecules were occasionally observed in the intermediate hydrophobic region (with Val206 and Phe136) between Glu138 and the BN center, illustrating a possible PT pathway directly from Channel 3.
4. *from PropD via Thr330 and Ser277*. This path along the sidechains of Thr330, Ser277, and Glu280 was originally proposed as a possible connection to the BN center in the X-ray structure [15]. It would be consistent with the experimental data which showed that conserved Glu280 and Glu211 are critical for both enzymatic activity and proton uptake and were suggested to directly participate in PT [21], [52], [63]. Thr330 and Ser277 are not protonatable residues and thus water molecules which could serve as proton sites are needed for PT along this path. Although water molecules are always present near PropD and there are no obstacles on the way to Thr330, they do not penetrate along this path, indicating that the cavity is very hydrophobic (Figures 7 and S9). During a 300 ns simulation a water molecule was found in the cavity only once for a very short time (Figure S11). Thus, from MD simulation it seems less likely that this path is used for the proton delivery to BN. Concerning the functional importance of Glu211 and Glu280, from the available cNOR structure it might be implied that Glu211Ala mutation probably destroys the coordination of Fe_B_, while Glu280 strongly affects processes at the BN center through electrostatics.

### No water channels from cytoplasm

**Figure 8:**
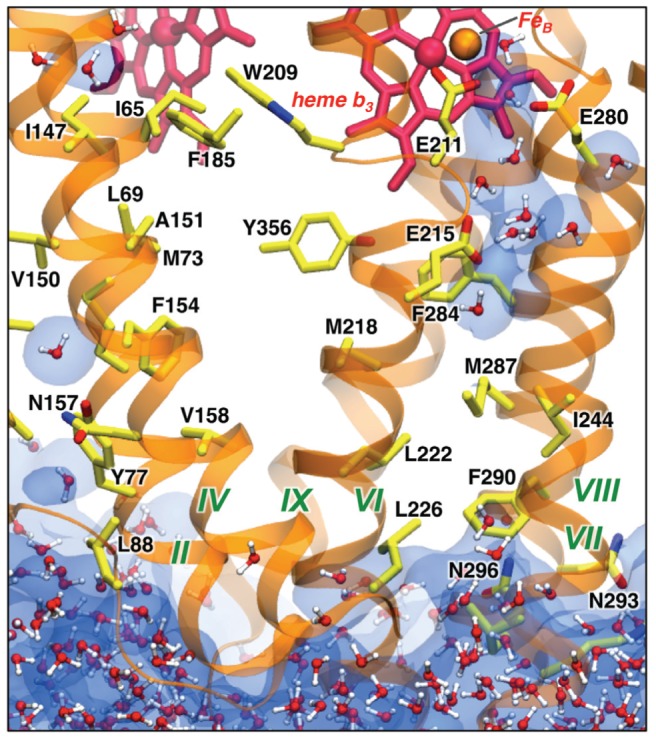
No water channels from the cytoplasm are found in cNOR. The region similar to the K- and D-pathways in oxidases is shown (MD snapshot at ∼100 ns). In cNOR, residues in this region are mostly hydrophobic. The interior region remains minimally hydrated (as shown by the water density averaged over 300 ns), except for a small charged region below the active site with Glu211, Glu280, and Glu215.

High-resolution crystal structures of CcO and subsequent mutational studies identified a number of critical residues in the proton pathways from the cytoplasm to the active site (K and D channels). However, in cNOR most of these residues are replaced by hydrophobic residues. The crystal structure of cNOR neither provides an obvious water channel from the cytoplasmic side of the membrane nor a H-bonded network in the regions that correspond to the K and D proton channels in CcO (see Figure 4 in ref. [15]). Similarly, our MD simulation shows no water in those regions (Figure 8), with the exception of a hydrophilic cavity below the active site with three glutamates, Glu211, Glu280, and Glu215. Thus, in cNOR there is no proton pathway from the cytoplasmic side. This is consistent with the experimental observations that cNOR is not electrogenic and has no proton-pumping activity, and that the electrons and protons for the catalytic reaction are supplied from the periplasmic side. The position of the above-mentioned small hydrophilic region overlaps with the terminal part of the K-pathway in cytochrome oxidases. That could indicate a beginning of the K-channel formation in the evolutionary steps leading to the appearance of proton pathways from the cytoplasm and eventually to the proton pumping in other HCOs. A very recent structural characterization of a single-subunit quinol-dependent NOR (qNOR) from *G. stearothermophilus* [64] surprisingly revealed the existence of the water channel from the cytoplasmic side at the position equivalent to the canonical K-pathway and absence of the periplasmic pathways found in cNOR. It will be interesting to test by calculations if a similar cytoplasmic channel can be formed in cNOR as the result of selective mutations.

### Concluding remarks

We have performed a 300 ns MD simulation of cNOR, based on its first crystal structure, and fully characterized water inside the protein. Our simulations have revealed two potential PT pathways from the periplasmic side, Channels 1 and 3. Both pathways are supported by the continuous distribution of water molecules and formation of the dynamic H-bonded networks within the channels, as well as by the highly conserved nature of the participating residues and previous experiments, which had shown functional importance of some of these residues. Since cNOR is not involved in a vectorial proton translocation (pumping against the gradient), a robust gating mechanism, as those suggested in CcO [56], [57], [62], [65], [66], is not required, and chemical protons have to arrive at the active site in one way or another. So, in principle, both pathways may be used. From our MD results we cannot unambiguously establish what the exact role of each channel is or how they are synchronized. In our opinion, Channel 1 is probably the main pathway for the proton uptake since both static and dynamic structures clearly show extensive H-bonded networks and water chains, and the path toward the catalytic site seems to be more straightforward. Meanwhile, Channel 3 is revealed only by the dynamic simulations (and the water channel is formed only for a part of the simulation), some protein structural rearrangements are required there to allow for channel formation, and the path from Glu138 to the active site goes through an intermediate hydrophobic region.

A further discussion about the details of the proton uptake mechanism in cNOR should be based on additional experimental evidences and explicit PT calculations. We would like to emphasize that MD simulations provide important information about the dynamics of water molecules and H-bonded networks and, as a result, about *locations of potential proton pathways*. However, classical MD simulations alone cannot describe explicit proton translocation, which is an intrinsically quantum mechanical process. The *energetics of PT* along different pathways has to be addressed by mixed QM/MM methods [25], [56], [67]–[69], and this will tell whether each pathway is feasible. The key issues in such calculations are the energies of charge formation at different sites along the translocation path and activation barriers of individual PT steps.

In our calculations we observed a fairly high number of mobile water molecules (which could not be resolved in the X-ray structure) in the cNOR hydrophilic cavities. Similar results were previously reported in analogous MD studies (with explicit membrane/solvent, at ambient temperatures) of systems like proton pumps cytochrome c oxidase [39], bacteriorhodopsin [28], [70], bc1 [71], voltage-gated proton channel Hv1 [27], [72] and calcium pump [73], [74], whose function relies on the water-assisted proton translocation. Therefore such simulations, although they are computationally expensive, can be used for the detailed characterization of water inside membrane proteins and for the identification of potential proton pathways, which in many cases are critical for protein function.

Finally, several common structural features, namely the position of the Ca^2+^ binding site and similarity of Channel 3 in cNOR and the periplasmic cavity in *cbb*_3_ oxidase, indicate the evolutionary relationship between the two enzymes. The likely loss of Channel 1 in *cbb*_3_ oxidase might be the key step during the molecular evolution leading to the establishment of the PT pathway from the cytoplasm, while a less effective Channel 3 was probably kept as a proton exit pathway for proton pumping. Our results have implications on the development of PT pathways in HCOs and the evolution of respiratory enzymes in general – a topic which remains a subject of intense debate.

## Supporting Information

Figure S1 Analysis of the MD trajectories. (**A**) The root-mean-square deviations (RMSD) of C_α_ atoms and (**B**) the root-mean-square fluctuations (RMSF) calculated for each residue with respect to the crystal structure. (**C**) Area/lipid, which was calculated using the Voronoi analysis tool, remains close to the experimental value for the POPE lipids (∼56 Å^2^) indicating a stable simulation of the protein-membrane complex.
(TIF)

Figure S2 (**A**) Entrance to Channel 1 (as viewed from the outside bulk). The protein is shown as a grey surface, water molecules as blue spheres, and lipid molecules as green sticks. A close-up view of the channel entrance is shown at the right side. The entrance site is formed by the amino acids Glu57_c_, Lys53_c_, Arg134, and Asp198. The Asp198 residue was assumed to be on the protein surface, but in the MD simulation it stays buried deeper inside the cavity. The entrance region remains rigid due to three stable salt bridges: Glu57_c_-Lys53_c_, Arg134-Asp198, and Lys53_c_-Asp198 (see Figure S3). A sidechain of a nearby Asn191, which is located in the bulk, exhibits large-amplitude rotations and mediates solvent molecules from the bulk into the channel cavity. (**B**) Solvent-accessible surface area (SASA) calculated for the residues of the Channel 1 entrance region. Average SASA values (from 31 frames, i.e. each 10 ns) are shown in brackets in the legend box.
(TIF)

Figure S3 Four stable salt bridges formed in Channel 1 in the course of the MD simulation. Top to bottom: time series of the distances between charged groups of Asp198-Arg134, Asp198-Lys53_c_, Glu57_c_-Lys53_c_, and Lys199-Glu70_c_. Black lines represent running averages over 30 data points.
(TIF)

Figure S4 Water molecules crossings through the entrance site of Channel 1. (**A**) Positions of five selected water molecules, which were observed crossing the Lys53_c_/Glu57_c_/Asp198 site during MD simulation, are shown as purple dots. (**B**) MD snapshot (after ∼20 ns) with a water molecule between the residues of the Channel 1 entrance site.
(TIF)

Figure S5 Representative configurations of the hydrogen-bonded networks in Channel 1. From left to right, then top to bottom: MD snapshots at 20, 53, 76, 108, 177, and 277 ns. The residues and color coding are the same as in Figure 2a in the main text. Due to dynamic properties of water molecules in Channel 1, the forming H-bonded networks are constantly “fluctuating”.
(TIF)

Figure S6 Time-series of the dihedral angles of the sidechains of (**A**) Glu138 (dihedral angle CA-CB-CG-CD) and (**B**) Asn60_c_ (dihedral angle CA-CB-CG-ND) in the MD simulation. Both sidechains show rotational flexibility: Glu138 takes three different conformations during the simulation, while the sidechain of Asn60_c_ is highly fluctuating, especially when the gate is open.
(TIF)

Figure S7 The Asn54-Asn60_c_ gate opening/closing events were also observed in the extended simulation (**A**) as well as in a short independent run (with different initial conditions) (**B**), indicating that such structural rearrangements can occur in cNOR on a 100-ns timescale.
(TIF)

Figure S8 Time series of the number of water molecules found near the active site (within 7 Å of both irons of the BN center). In contrast to the crystal structure, the MD simulation reveals the presence of water molecules near the BN center.
(TIF)

Figure S9 Time series of the distances to Fe_B_ of several selected water molecules (each colored line represents one water molecule). Water molecules near the BN center keep their positions at the “permanent” water sites much longer than mobile waters in Channel 1.
(TIF)

Figure S10 Water density in the region near the active site (including the terminal parts of Channels 1 and 3), shown as a 2D contour map. The water density was averaged over 300 ns. Positions of the important residues and two hemes (shown as sticks) and crystallographic waters (purple spheres) in the cNOR X-ray structure are superimposed on the contour map for reference. Possible pathways for the final PT steps to the BN center are indicated by the dashed black lines and marked with numbers 1 to 4 (see discussion in section *PT pathways near the active site* in the main text).
(TIF)

Figure S11 A single snapshot (at ∼260 ns) when a water molecule was found in the hydrophobic cavity between PropD and Thr330.
(TIF)

Table S1 Involvement of the Channel 1 residues in the formation of the H-bonded networks.
(DOCX)

Text S1 Details of the MD system setup, simulation and analysis protocols.
(DOCX)

Video S1 Hydrogen-bonded networks in Channel 1. Movie illustrates the dynamics in Channel 1 along a 300 ns MD trajectory. In particular it shows a regular formation of the dynamic hydrogen-bonded water chains. The residues and color coding are the same as in Figure 2a. Only water molecules present in or near Channel 1 are shown. Several snapshots of this movie are presented in Figure S4.
(AVI)

Video S2 Gating of Channel 3. Movie (a 120–260 ns part of the MD trajectory) illustrates the opening/closing of the Asn54-Asn60_c_ gate and formation of a new water channel. The residues and color coding are the same as in Figure 4a in the main text. Only water molecules present in or near Channel 3 are shown.
(AVI)
