# Supplementary figures and images for: Molecular Dynamics Simulations Reveal Proton Transfer Pathways in Cytochrome C-Dependent Nitric Oxide Reductase
Source: PLoS Comput Biol. 2012 Aug 30;8(8):e1002674. doi: 10.1371/journal.pcbi.1002674 (PMC3431322; doi:10.1371/journal.pcbi.1002674)

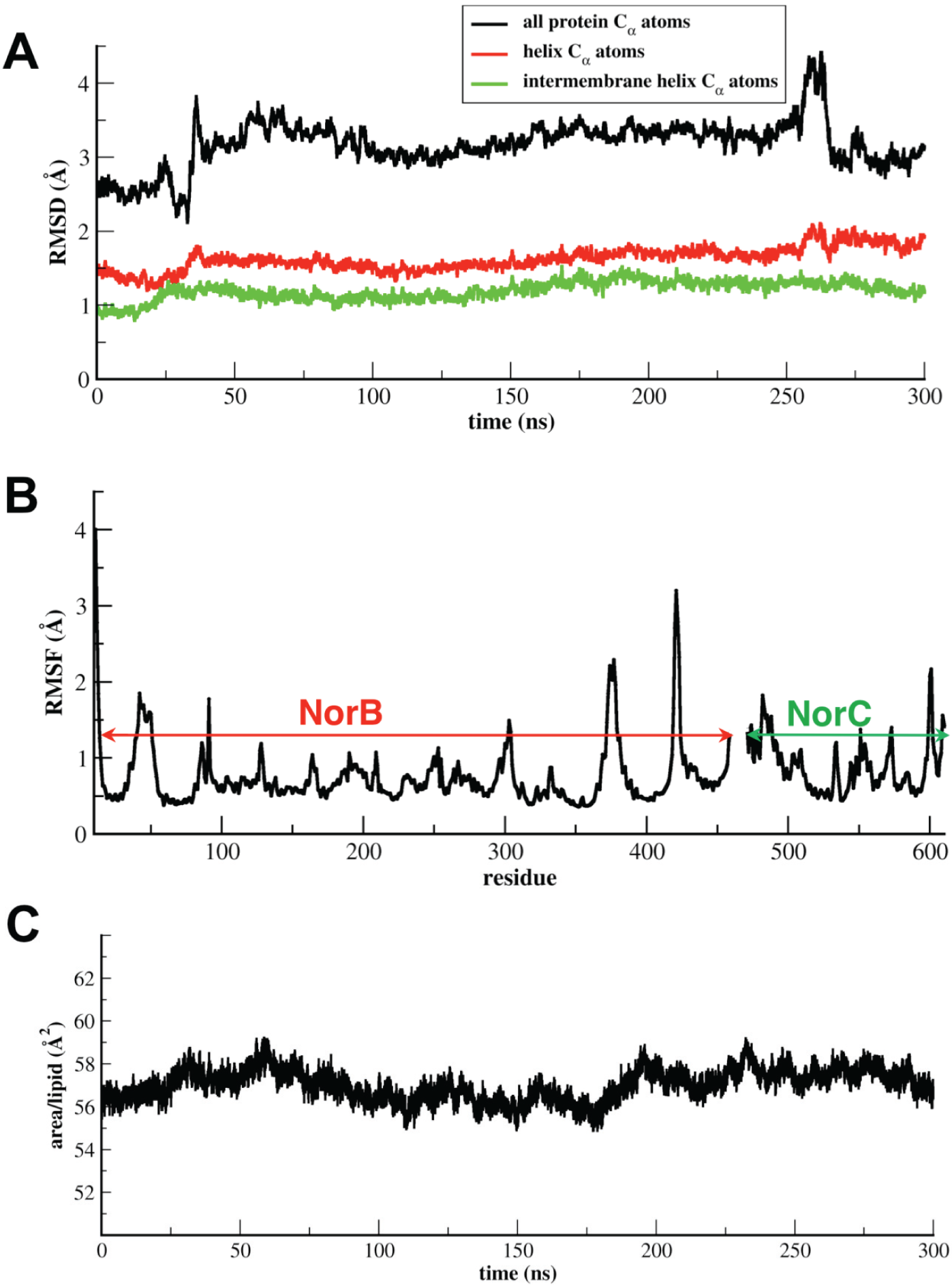

Supplement: Figure S1 — Analysis of the MD trajectories. (A) The root-mean-square deviations (RMSD) of Cα atoms and (B) the root-mean-square fluctuations (RMSF) calculated for each residue with respect to the crystal structure. (C) Area/lipid, which was calculated using the Voronoi analysis tool, remains close to the experimental value for the POPE lipids (∼56 Å2) indicating a stable simulation of the protein-membrane complex. (TIF) [file pcbi.1002674.s001.tif]

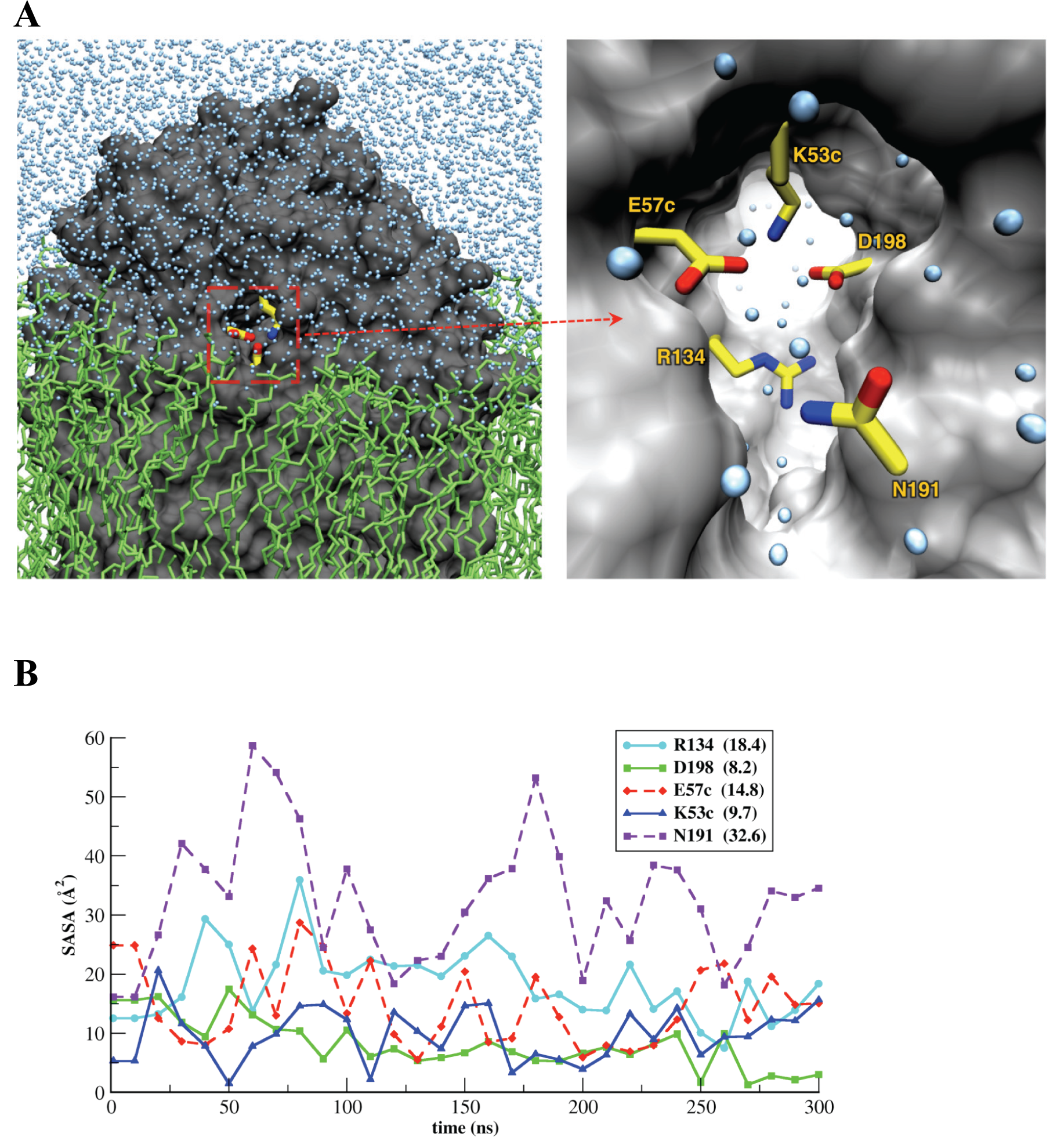

Supplement: Figure S2 — (A) Entrance to Channel 1 (as viewed from the outside bulk). The protein is shown as a grey surface, water molecules as blue spheres, and lipid molecules as green sticks. A close-up view of the channel entrance is shown at the right side. The entrance site is formed by the amino acids Glu57c, Lys53c, Arg134, and Asp198. The Asp198 residue was assumed to be on the protein surface, but in the MD simulation it stays buried deeper inside the cavity. The entrance region remains rigid due to three stable salt bridges: Glu57c-Lys53c, Arg134-Asp198, and Lys53c-Asp198 (see Figure S3). A sidechain of a nearby Asn191, which is located in the bulk, exhibits large-amplitude rotations and mediates solvent molecules from the bulk into the channel cavity. (B) Solvent-accessible surface area (SASA) calculated for the residues of the Channel 1 entrance region. Average SASA values (from 31 frames, i.e. each 10 ns) are shown in brackets in the legend box. (TIF) [file pcbi.1002674.s002.tif]

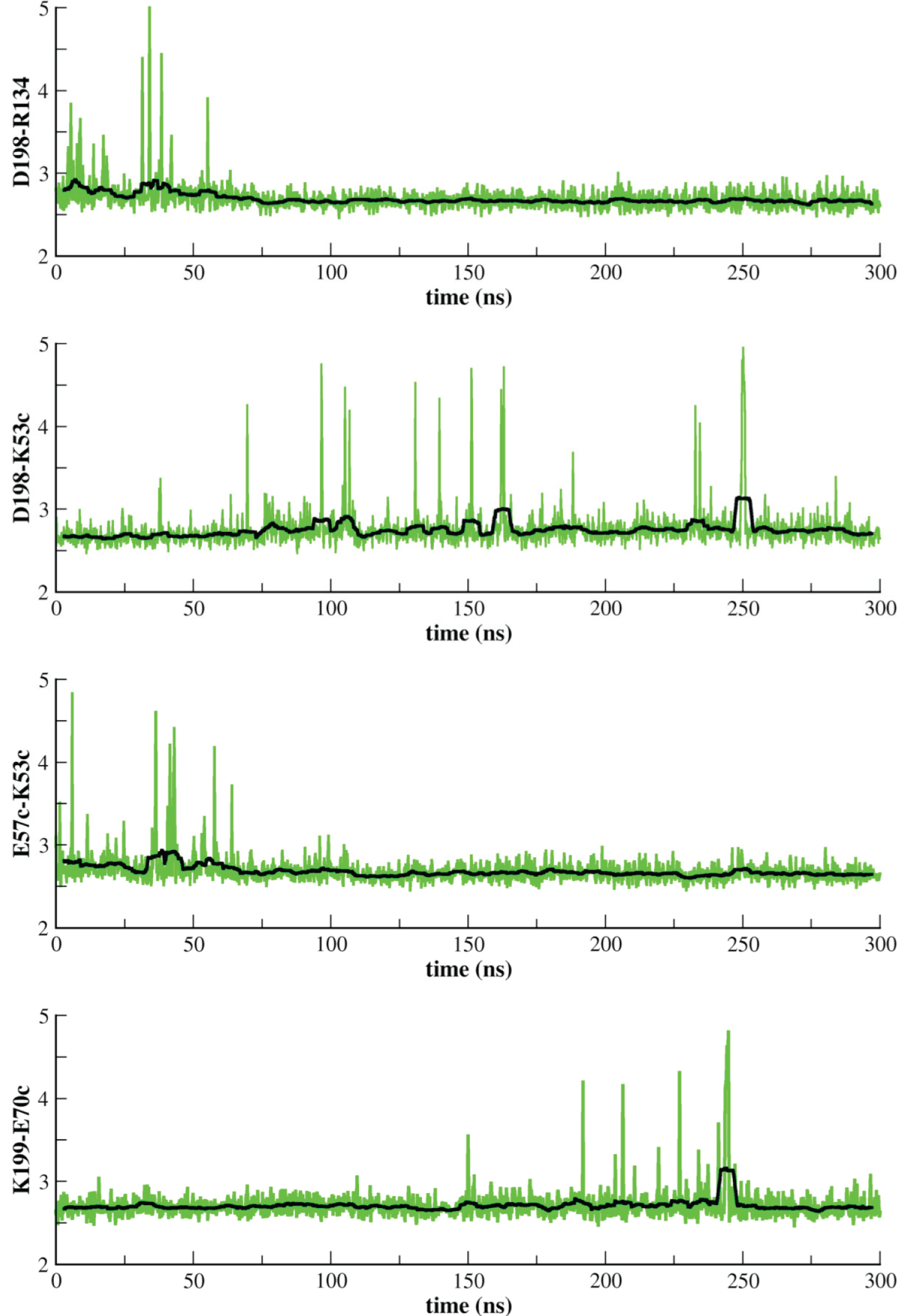

Supplement: Figure S3 — Four stable salt bridges formed in Channel 1 in the course of the MD simulation. Top to bottom: time series of the distances between charged groups of Asp198-Arg134, Asp198-Lys53c, Glu57c-Lys53c, and Lys199-Glu70c. Black lines represent running averages over 30 data points. (TIF) [file pcbi.1002674.s003.tif]

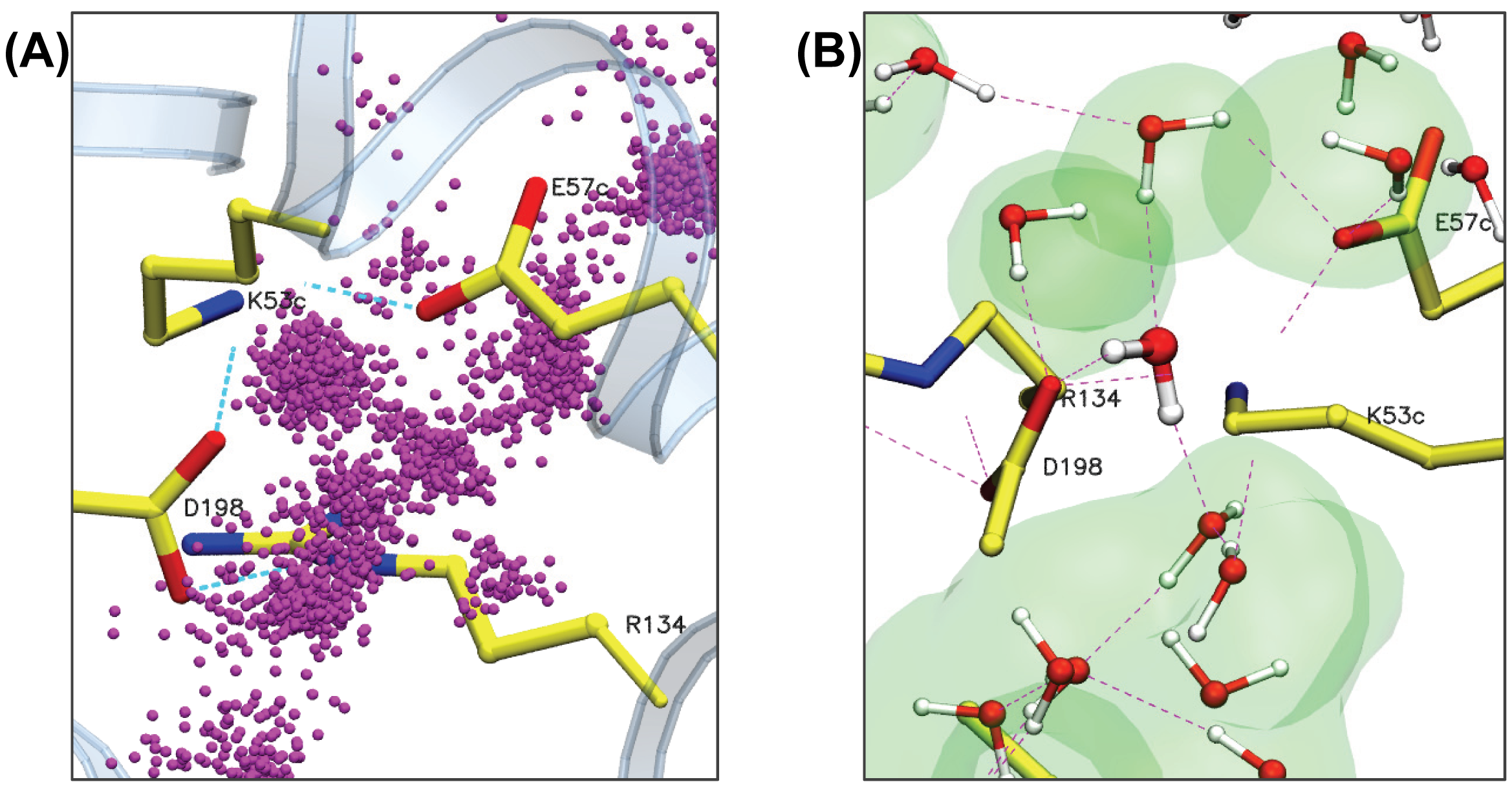

Supplement: Figure S4 — Water molecules crossings through the entrance site of Channel 1. (A) Positions of five selected water molecules, which were observed crossing the Lys53c/Glu57c/Asp198 site during MD simulation, are shown as purple dots. (B) MD snapshot (after ∼20 ns) with a water molecule between the residues of the Channel 1 entrance site. (TIF) [file pcbi.1002674.s004.tif]

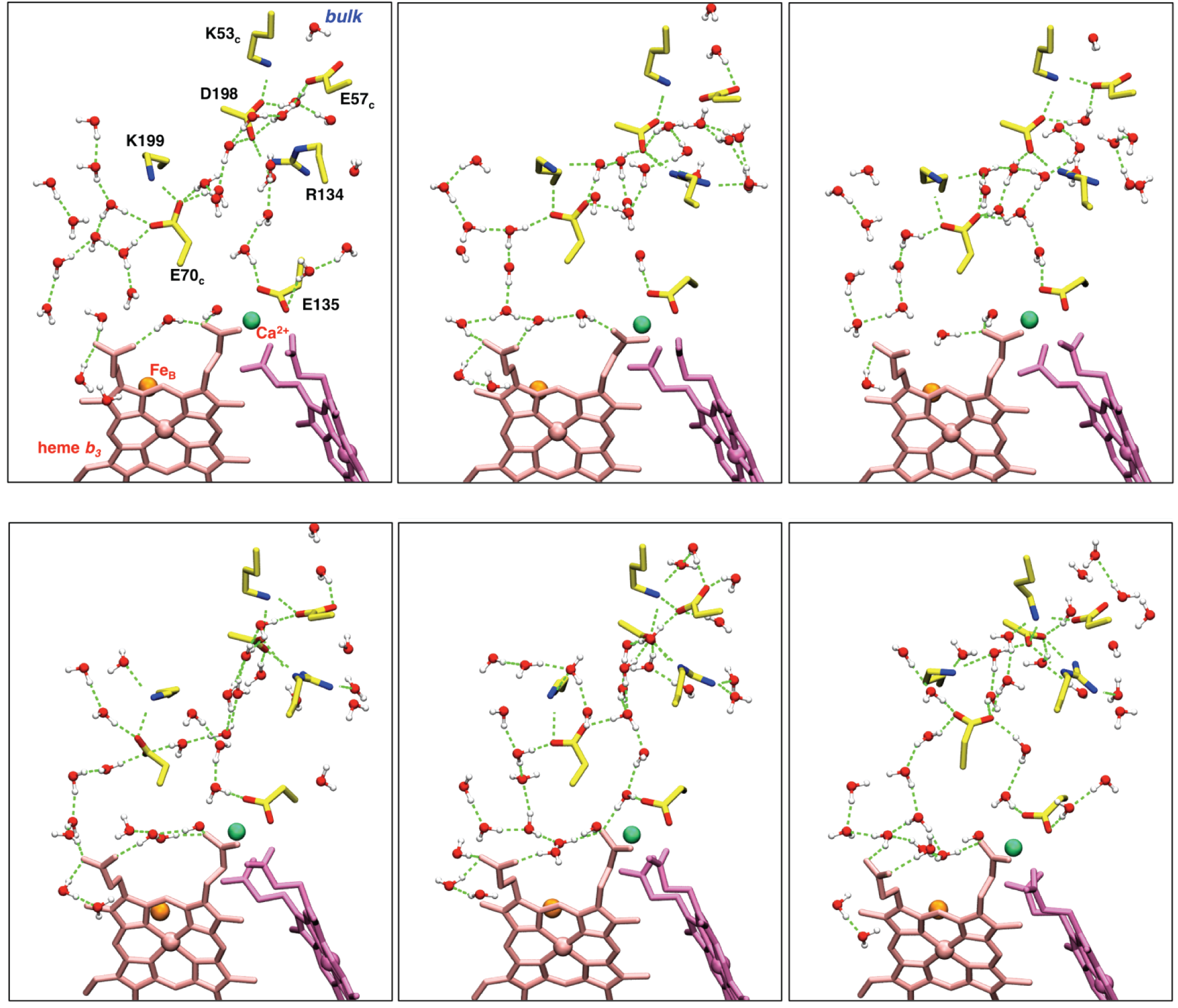

Supplement: Figure S5 — Representative configurations of the hydrogen-bonded networks in Channel 1. From left to right, then top to bottom: MD snapshots at 20, 53, 76, 108, 177, and 277 ns. The residues and color coding are the same as in Figure 2a in the main text. Due to dynamic properties of water molecules in Channel 1, the forming H-bonded networks are constantly “fluctuating”. (TIF) [file pcbi.1002674.s005.tif]

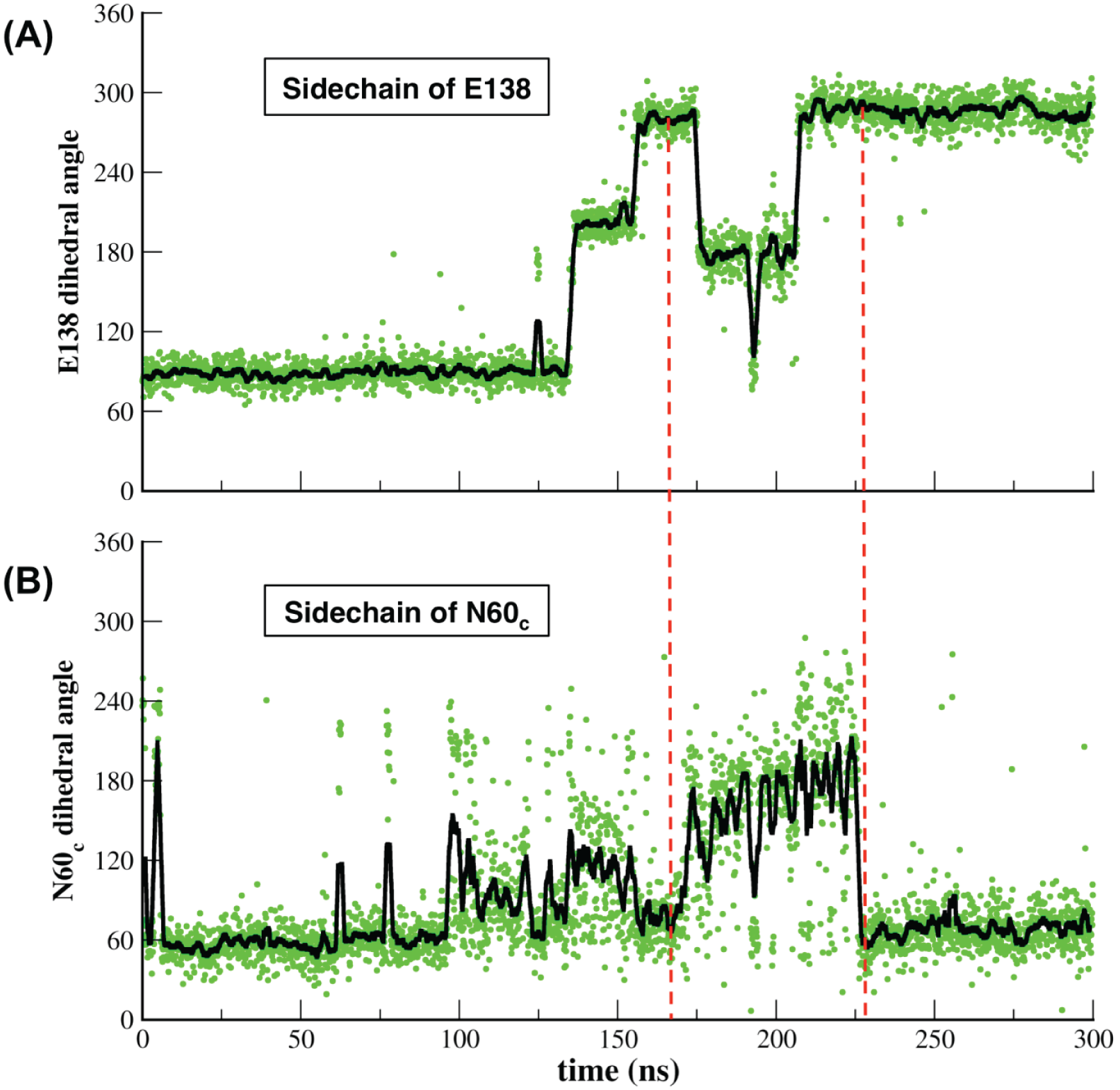

Supplement: Figure S6 — Time-series of the dihedral angles of the sidechains of (A) Glu138 (dihedral angle CA-CB-CG-CD) and (B) Asn60c (dihedral angle CA-CB-CG-ND) in the MD simulation. Both sidechains show rotational flexibility: Glu138 takes three different conformations during the simulation, while the sidechain of Asn60c is highly fluctuating, especially when the gate is open. (TIF) [file pcbi.1002674.s006.tif]

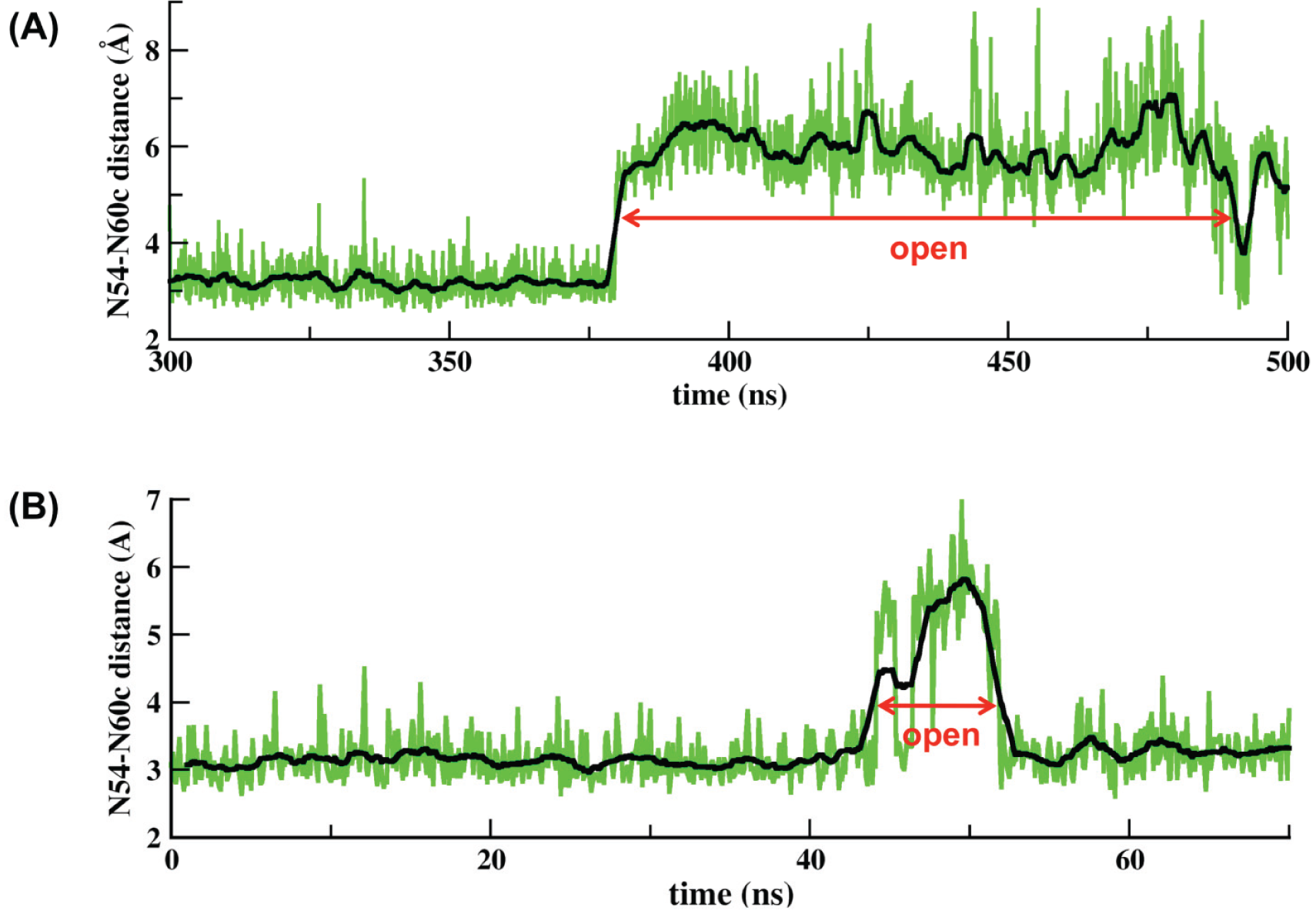

Supplement: Figure S7 — The Asn54-Asn60c gate opening/closing events were also observed in the extended simulation (A) as well as in a short independent run (with different initial conditions) (B), indicating that such structural rearrangements can occur in cNOR on a 100-ns timescale. (TIF) [file pcbi.1002674.s007.tif]

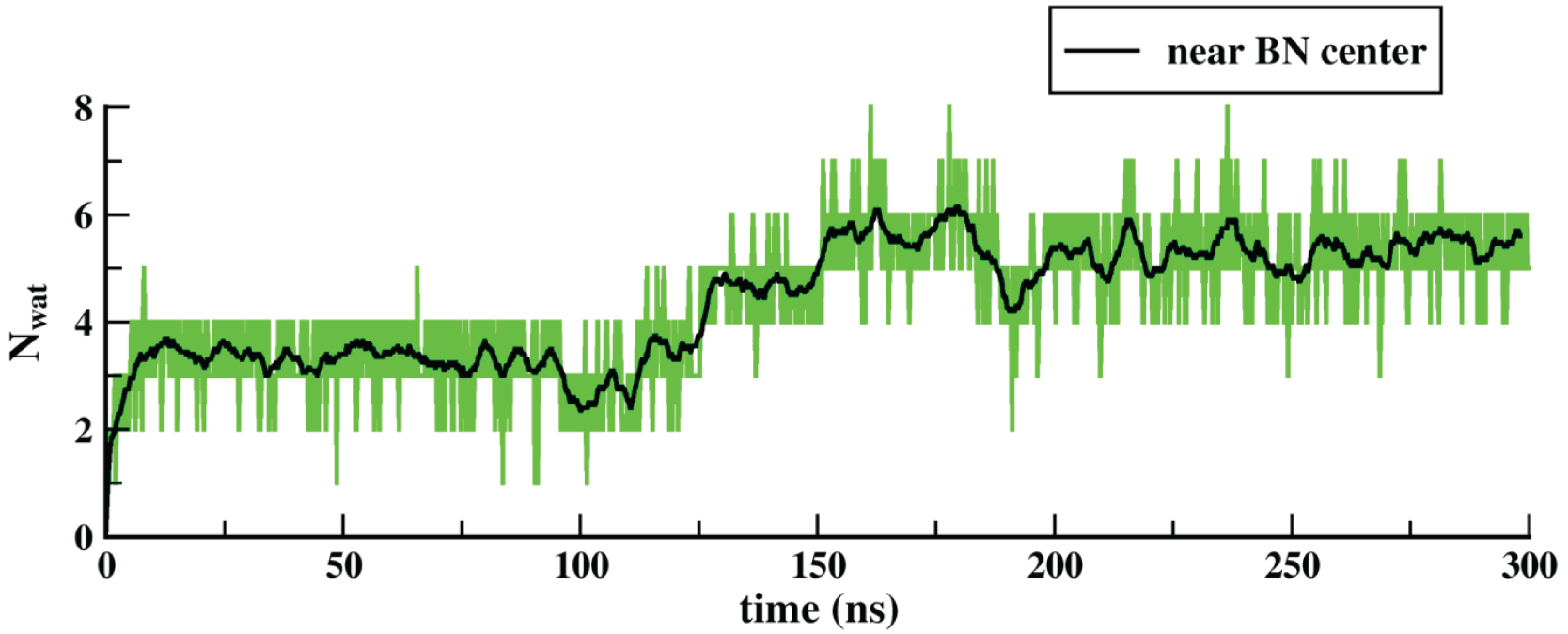

Supplement: Figure S8 — Time series of the number of water molecules found near the active site (within 7 Å of both irons of the BN center). In contrast to the crystal structure, the MD simulation reveals the presence of water molecules near the BN center. (TIF) [file pcbi.1002674.s008.tif]

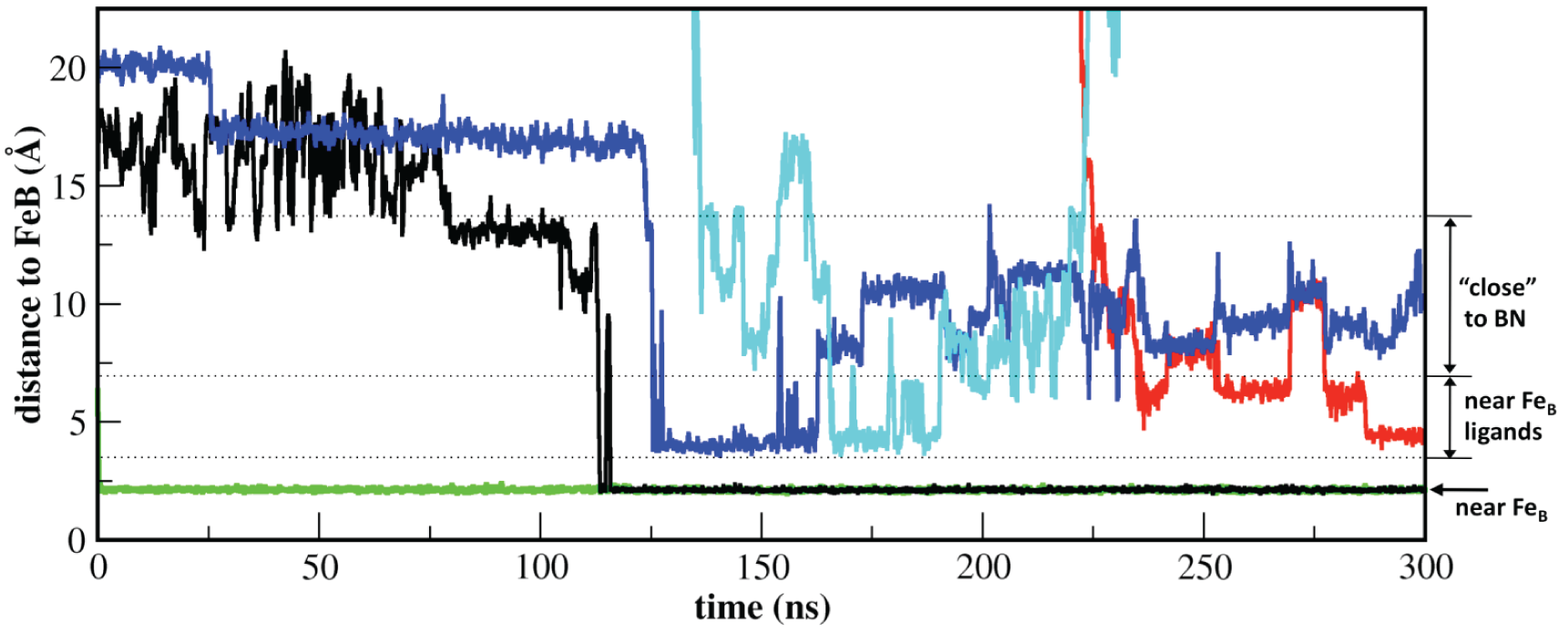

Supplement: Figure S9 — Time series of the distances to FeB of several selected water molecules (each colored line represents one water molecule). Water molecules near the BN center keep their positions at the “permanent” water sites much longer than mobile waters in Channel 1. (TIF) [file pcbi.1002674.s009.tif]

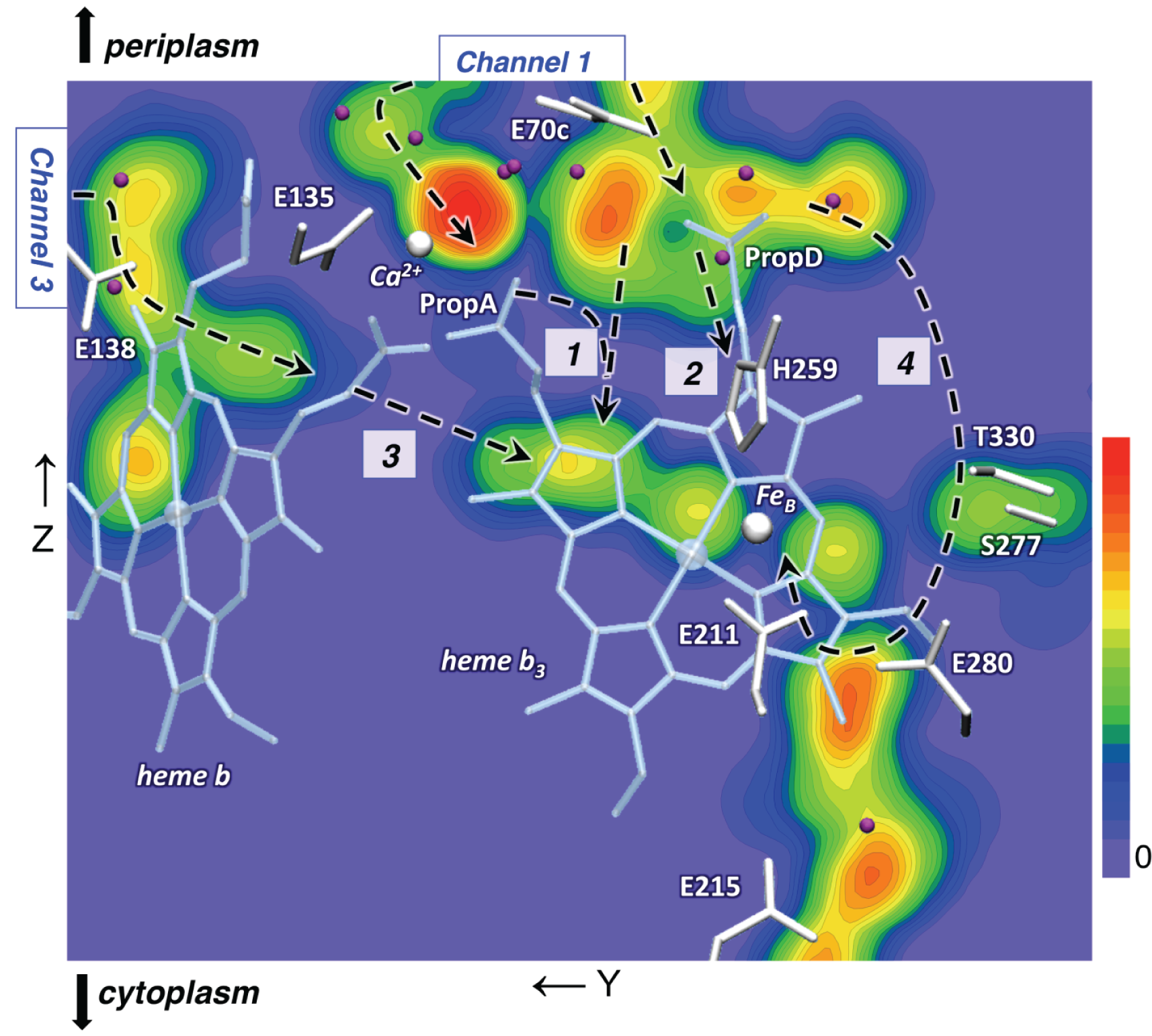

Supplement: Figure S10 — Water density in the region near the active site (including the terminal parts of Channels 1 and 3), shown as a 2D contour map. The water density was averaged over 300 ns. Positions of the important residues and two hemes (shown as sticks) and crystallographic waters (purple spheres) in the cNOR X-ray structure are superimposed on the contour map for reference. Possible pathways for the final PT steps to the BN center are indicated by the dashed black lines and marked with numbers 1 to 4 (see discussion in section PT pathways near the active site in the main text). (TIF) [file pcbi.1002674.s010.tif]

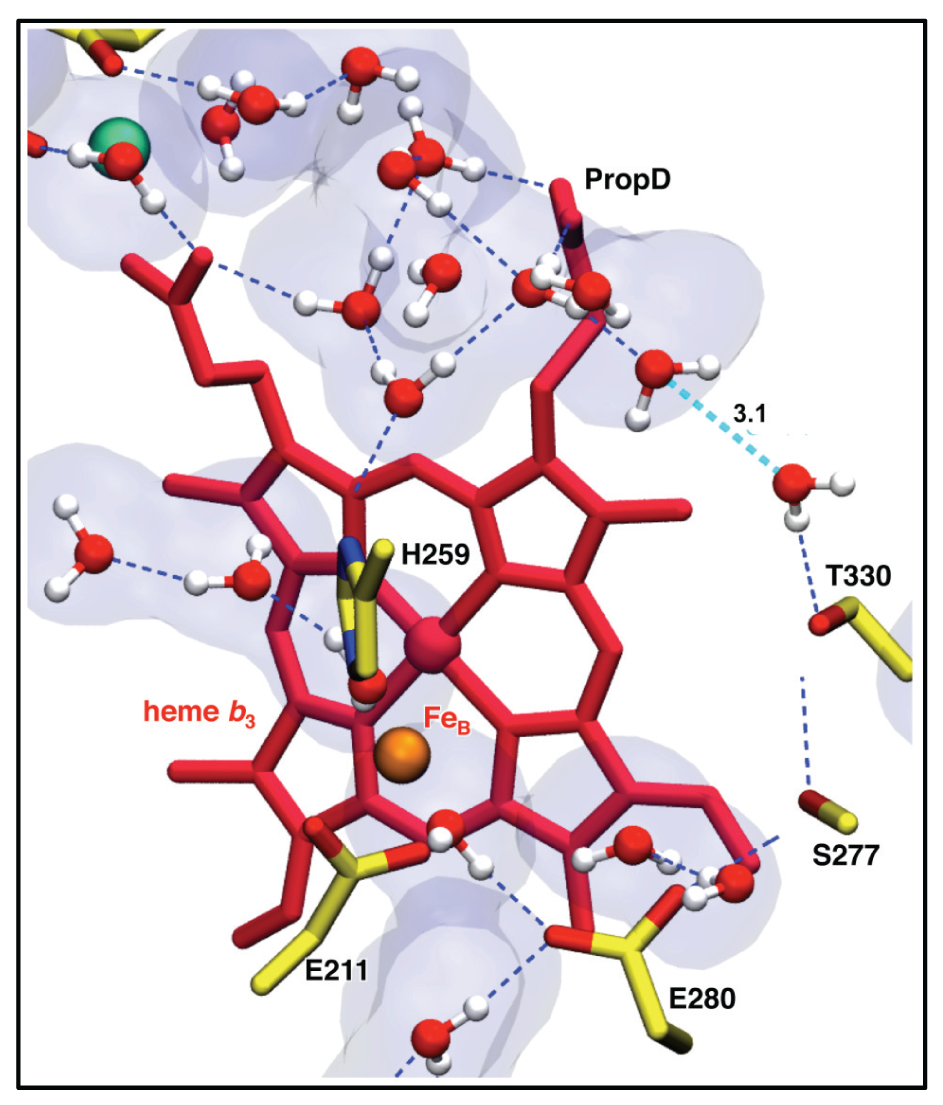

Supplement: Figure S11 — A single snapshot (at ∼260 ns) when a water molecule was found in the hydrophobic cavity between PropD and Thr330. (TIF) [file pcbi.1002674.s011.tif]
