# Supplementary material for: Molecular Dynamics Simulations Reveal Proton Transfer Pathways in Cytochrome C-Dependent Nitric Oxide Reductase
Source: PLoS Comput Biol. 2012 Aug 30;8(8):e1002674. doi: 10.1371/journal.pcbi.1002674 (PMC3431322; doi:10.1371/journal.pcbi.1002674)
Supplement: Table S1 — Involvement of the Channel 1 residues in the formation of the H-bonded networks. (DOCX) [file pcbi.1002674.s012.docx]

**Table S1:** Involvement of the Channel 1 residues in the formation of the H-bonded networks.

| residue | <N_wats_> | <N_HBs_> |
| --- | --- | --- |
| Glu135 | 3.8 | 1.3 |
| Arg134 | 6.5 | 2.2 |
| Asp198 | 4.0 | 2.3 |
| Lys199 | 4.9 | 1.2 |
| Glu70_c_ | 7.0 | 3.6 |
| Glu57_c_ | 5.5 | 2.6 |
| Lys53_c_ | 3.4 | 0.5 |

<N_wats_> is the average number of water molecules around each residue sidechain. <N_HBs_> is the average number of HBs formed between each residue and nearby water molecules.
