# Supplementary material for: Molecular Dynamics Simulations Reveal Proton Transfer Pathways in Cytochrome C-Dependent Nitric Oxide Reductase
Source: PLoS Comput Biol. 2012 Aug 30;8(8):e1002674. doi: 10.1371/journal.pcbi.1002674 (PMC3431322; doi:10.1371/journal.pcbi.1002674)
Supplement: Text S1 — Details of the MD system setup, simulation and analysis protocols. (DOCX) [file pcbi.1002674.s013.docx]

**Molecular dynamics simulations reveal proton transfer pathways**

**in cytochrome c-dependent nitric oxide reductase**

Andrei V. Pisliakov, Tomoya Hino, Yoshitsugu Shiro, and Yuji Sugita

**Supporting Information**

**MD simulation setup**

The simulations of cNOR were set up as follows. The initial system was prepared from a 2.7 Å resolution crystal structure (PDB ID: 3O0R) of the cNOR enzyme from *Ps. aeruginosa* [1]. That structure was solved in complex with the antibody fragment (Fab), which is positioned in the bulk above the hydrophilic domain of the NorC subunit, far away from the water channels discussed in this work. The Fab fragment was not included in the simulations. The residues missing in the crystal structure (termini regions) were added with the MODELLER program [2]. All crystallographic water molecules of the NorB and NorC subunits were kept, and additional water molecules were added in the protein hydrophillic pockets with the Dowser program [3]. Titratable residues of the pathways discussed in the text were predicted to be in their default states at pH 7, as determined by pK_a_ calculations using MEAD [4]. Therefore, in MD simulation all charged residues were kept at their standard protonation states.

The cNOR structure [1] corresponds to the mixed redox state: the crystal was prepared in the oxidized state; however, during the X-ray data collection it was partially reduced as indicated by the optical absorption spectra. Here cNOR was modeled in a reduced state. A single oxygen atom resolved between two irons of the BN center (presumably, a μ-oxo-bridge ligand in the resting state) was removed.

A lipid bilayer membrane was constructed of 352 POPE (palmitoyl-oleoyl-phosphatidylethanolamine) lipid molecules and was initially equilibrated for 100 ns to produce thickness and area/lipid that correspond to experimental values. The membrane normal was oriented along the vertical Z axis. The protein was embedded into the equilibrated lipid bilayer membrane and aligned *vs* membrane by using the OPM algorithm [5], which resulted in a tilt angle of 7.2±1.7°. The lipid molecules overlapping with the protein atoms were removed. A protein-membrane complex was fully solvated in a water box. Na^+^ and Cl^-^ ions at a 150 mM concentration were added to the system. The total size of the simulation system was 110,292 atoms and the simulation cell dimensions were 118x118x112 Å.

**Simulation protocol**

All MD simulations were carried out in NAMD (ver. 2.7b3) [6] with the CHARMM all-atom force field for proteins and lipids (ver. 27 with the CMAP protein backbone correction terms) [7,8] and the TIP3P model for water [9,10]. We used the available CHARMM parameters for the heme and suitable patches for specific heme coordinations [11]. The binuclear center (BNC) in cNOR (with Fe_B_ and additional Glu ligand, but no cross-linked His-Tyr) is significantly different from the canonical heme a_3_-Cu_B_ center in cytochrome c oxidase (CcO). Therefore, the parameters available for the Cu_B_ site in CcO (e.g. [12]) cannot be adopted in cNOR. MD charges of FeB and its ligands were scaled to account for charge delocalization. An accurate parametrization of such di-iron BNC is *per se* a challenging computational project, and is beyond the scope of our current work.

The system was subjected to a short minimization, followed by several equilibration runs (5 ns), with the constraints applied to the heavy protein atoms and lipid head groups. After equilibration, we performed 300 ns unconstrained production MD runs, which were used for analysis. The simulation later was extended to 500 ns, and the extended part (i.e. 300-500 ns) was used for additional analysis of the Channel 3 region (see Figure S6). Two short independent runs starting from different initial conditions were additionally performed for statistics. In the simulations, constant temperature (300 K), pressure (1 atm), and surface area were maintained (NPAT ensemble). The bond lengths to hydrogen atoms were constrained using the Shake algorithm, allowing a time step of 2 fs. To maintain the geometry of the Fe_B_ site close to the crystal structure, harmonic restraints (the ‘extrabonds’ feature in NAMD) with the force constant k=10 were applied to the bonds and angles between Fe_B_ and its ligands.

**Analysis protocol**

Snapshots of the MD trajectories were saved every 10 ps. VMD [13] with scripts developed in-house was used for the analysis of the trajectories. In the analysis of hydrogen bonds (HBs), a HB was defined using “easy” geometric criteria, namely a donor (D)/acceptor (A) distance <3.6 Å and a D-H…A angle >145°. Water densities were calculated using the VMD VolMap plugin, which produced volumetric maps (3D grids of values – in this case, the water oxygen occupancies) of the water molecules found in a specified region at each MD snapshot, which were then averaged over the MD trajectory. The generated water densities were visualized as (i) 3D isosurfaces (drawn at a 25% occupancy level, if not otherwise specified), showing the extent of the hydrophilic regions in the MD simulation, and (ii) 2D contour maps (which were produced by summing the generated water densities over a specified axis) using the Surfer (Golden Software, Inc.) program.

Molecular images in the main text and Supporting Information were prepared using PyMol [14] and VMD.

**References**

1. Hino T, Matsumoto Y, Nagano S, Sugimoto H, Fukumori Y, et al. (2010) Structural basis of biological N2O generation by bacterial nitric oxide reductase **Science** 330: 1670-1669.

2. Sali A, Blundell TL (1993) Comparative protein modeling by satisfaction of spatial restraints. **J Mol Biol** 234: 779-815.

3. Zhang L, Hermans J (1996) Hydrophilicity of cavities in proteins. **Proteins: Struct Funct Genet** 24: 433-438.

4. Bashford D, Gerwert K (1992) Electrostatic calculation of the pka values of ionizable groups in bacteriorhodopsin. **J Mol Biol** 224: 473-486.

5. Lomize AL, Pogozheva ID, Lomize MA, Mosberg HI (2006) Positioning of proteins in membranes: A computational approach. **Protein Sci** 15: 1318-1333.

6. Phillips JC, Braun R, Wang W, Gumbart J, Tajkhorshid E, et al. (2005) Scalable molecular dynamics with NAMD. **J Comput Chem** 26: 1781-1802.

7. MacKerell AD, Bashford D, Bellott M, Dunbrack RL, Evanseck JD, et al. (1998) All-atom empirical potential for molecular modeling and dynamics studies of proteins. **J Phys Chem B** 102: 3586-3616.

8. Mackerell AD, Feig M, Brooks CL (2004) Extending the treatment of backbone energetics in protein force fields: Limitations of gas-phase quantum mechanics in reproducing protein conformational distributions in molecular dynamics simulations. **J Comput Chem** 25: 1400-1415.

9. Jorgensen WL, Chandrasekhar J, Madura JD, Impey RW, Klein ML (1983) Comparison of simple potential functions for simulating liquid water. **J Chem Phys** 79: 926 - 935.

10. Neria E, Fischer S, Karplus M (1996) Simulation of activation free energies in molecular systems. **J Chem Phys** 105: 1902-1921.

11. Autenrieth F, Tajkhorshid E, Baudry J, Luthey-Schulten Z (2004) Classical force field parameters for the heme prosthetic group of cytochrome c. **J Comput Chem** 25: 1613-1622.

12. Johansson MP, Kaila VRI, Laakkonen L (2008) Charge parameterization of the metal centers in cytochrome c oxidase. **J Comput Chem** 29: 753-767.

13. Humphrey W, Dalke A, Schulten K (1996) VMD: Visual molecular dynamics. **J Mol Graph** 14: 33-38.

14. The PyMOL Molecular Graphics System V, Schrödinger, LLC.
